# Well-Being Adjusted Health Expectancy: A New Summary Measure of Population Health

**DOI:** 10.1007/s10680-022-09628-1

**Published:** 2022-08-08

**Authors:** Magdalena Muszyńska-Spielauer, Marc Luy

**Affiliations:** Vienna Institute of Demography (OeAW), Wittgenstein Centre for Demography and Global Human Capital (IIASA, OeAW, University of Vienna), Vienna, Austria

**Keywords:** Health expectancy, Health adjusted life expectancy, Well-being, Health, EU-SILC, Well-being adjusted life expectancy, WAHE

## Abstract

We propose a new summary measure of population health (SMPH), the well-being-adjusted health expectancy (WAHE). WAHE belongs to a subgroup of health-adjusted life expectancy indicators and gives the number of life years equivalent to full health. WAHE combines health and mortality information into a single indicator with weights that quantify the reduction in well-being associated with decreased health. WAHE's advantage over other SMPHs lies in its ability to differentiate between the consequences of health limitations at various levels of severity and its transparent, simple valuation function. Following the guidelines of a Committee on Summary Measures of Population Health, we discuss WAHE's validity, universality, feasibility sensitivity and ensure its reproducibility. We evaluate WAHE's performance compared to life expectancy, the most commonly used indicators of health expectancy (HE) and disability-adjusted life expectancy (DALE) in an empirical application for 29 European countries. Data on health and well-being are taken from the 2018 EU-SILC, and the life tables are from Eurostat. DALE is taken from the database of the Global Burden of Disease Programme. WAHE's sensitivity to univariate and multivariate state specifications is studied using the three Minimum European Health Module health dimensions: chronic morbidity, limitations in activities of daily living, and self-rated health. The empirical tests of the indicators’ correspondence reveal that WAHE has the strongest correlation with the other SMPHs. Moreover, WAHE estimates are in agreement with all other SMPHs. Additionally, WAHE and all other SMPHs form a group of reliable indicators for studying population health in European countries. Finally, WAHE estimates are robust, regardless of whether health is defined across one or multiple simultaneous dimensions of health. We conclude that WAHE is a useful and reliable indicator of population health and performs at least as well as other commonly used SMPHs.

**Supplementary Information:**

The online version contains supplementary material available at 10.1007/s10680-022-09628-1.

## Introduction

A summary measure of population health (SMPH) combines information on health and mortality into a single number. SMPHs most familiar to demographers are health expectancy (HE) and disability-adjusted life expectancy (DALE) from the Global Burden of Disease (GBD). Despite their widespread use, both HE and DALE have received frequent criticism from the scientific community. The aim of this article is to propose and validate a new SMPH that overcomes the best-known limitations of these two indices.

The first limitation of the two measures relates to the technical details of their construction. Because HE is based on a dichotomy of full and decreased health, a primary concern is its inability to distinguish between specific levels of disease severity. Additionally, such dichotomous distinctions between full and decreased health are based on arbitrary threshold definitions and hence influence study outcomes and conclusions (Murray et al., 2002). DALE overcomes these dichotomies and arbitrary thresholds limitations by including information on different health states. Nevertheless, it has been criticised for its solutions to construct weights for the health states, differential age weighting, and discounting procedures (Anand & Hanson, 1997; Voigt & King, 2014). Although, since its development in the early 1990s, age-weighting and discounting in DALE have been discontinued and valuation of health states, which were originally collected from a panel of health care professionals, is today conducted based on population surveys (Salomon et al., 2015, Solberg et al., 2020), the process of obtaining disability weights and values of the weights are still questioned (Nord, 2013, 2015). An additional limitation of both, HE and DALE, refers to the incomparability of their levels across different populations or time, that is, cross-contextual invalidity of the two indices. None of the indices takes into account that the concept of health status is context-specific: Social and medical contexts shape peoples' awareness of health problems and influence the consequences of decreased health (Anand & Hanson, 1997; Voigt & King, 2014), but also self-assessment of health status (Jürges, 2007).

The new measure we propose in this article is based on recent developments in health valuation in health economics and analogous to the one proposed recently by the philosopher Hausman (2015), who suggested that weighting health states by well-being would better accomplish the purpose of SMPH to compare health across populations. We refer to this new indicator as *well-being adjusted health expectancy* (WAHE). This term was chosen to reflect technical solutions in constructing the new measure: To base the valuation function for different health states on the subjective well-being of those who experience a given health state. This valuation is also context-specific, which means that it can be estimated separately depending on the selected contextual-level characteristics. We propose solutions to derive the well-being weights and the new indicator. Next, following the guidelines of the Committee on Summary Measures of Population Health (Gold & Field, 1998), we validate the proposed indicator and compare it against other SMPHs in a theoretical discussion and an empirical application. The validation in the empirical application follows the guidelines of quality diagnostics of newly developed classifications, scales, and other measurement instruments in clinical studies (Kottner et al., 2011).

## Derivation of the WAHE Indicator

### The General Formula

Health expectancy (HE) and health-adjusted life expectancy (HALE)—including the commonly used indicators DALE and quality-adjusted life years (QALY)—quantify the average number of live years spent in good health in a stationary population.^1^ Both measures are constructed based on the same formula, while they differ in the number of distinguished and valued health states: HE is based on a dichotomous health state definition that simply divides the number of years lived into those spent in good and decreased health states. Conversely, HALE defines health across a discrete number of states, with decreased health being defined across more than one state. The life years are then combined into a single indicator with weights according to the degree of dysfunction they are lived with. While HE quantifies the average number of years lived in good health in a stationary population, HALE measures the average number of years equivalent to full health (Mathers, 2002). HALE's advantage over HE lies in its technical ability to account for the severity of health impairments and incorporates the information about the specific value of decreased health states compared to full health. Analogous to life expectancy (LE) derived from a life table, HALE at age $x$ is calculated based on the number of years lived in specific health states at this age and above as:

1 $${HALE}_{x}=\frac{1}{{l}_{x}}\sum_{k=1}^{n}\sum_{a=x}^{\omega }{\pi }_{k,a}{L}_{k,a}$$

where ${l}_{x}$ is the number of life table survivors at age $x$, ${L}_{k,a}$ is the number of years lived in health state $k$ at age $a$, and ${\pi }_{k,a}$ is the health-related weight assigned to the health state $k$ at age $a$. The HALE weights indicate the relative value of a year spent in a given health state $k$ compared to a year spent in full health at age $a$ (Mathers, 2002). By contrast, following the above formula, HE considers only two health states ($k=\mathrm{1,2}$), a single state of full health with weight ${\pi }_{k,a}=1$ for all age groups and a single state of decreased health with a zero weight $({\pi }_{k,a}=0)$.

### Life Years Lived in Different Health States

Years lived in different health states can be estimated with the method developed by Sullivan (1971), double-decrement life table or multistate life table methods. We discuss in detail the application of the Sullivan method, as it is the most commonly used formula to estimate SMPH, due to its low computational and data requirements. While the other two methods are based on transition rates between various health states obtained from panel surveys, the Sullivan method requires only cross-sectional data (Jagger & Robine, 2011; Rogers et al., 1990). Despite these different approaches, Mathers and Robine (1997) have shown that they yield comparable results, unless health transitions rates are subject to rapid and substantial changes.

The Sullivan method combines the number of years lived from a period life table with the health state prevalence from cross-sectional survey data. The number of years lived at age *a* in health state *k* ($${L}_{k,a})$$ is simply derived by redistributing the total number of years lived at age *a* ($${L}_{a})$$ according to the prevalence of this state at age *a* in the surveyed population $${(h}_{k,a})$$ as $${L}_{k,a}={h}_{k,a} {L}_{a}$$.

When based on the Sullivan method, the WAHE indicator is estimated from the general HALE formula (Formula 1) by weighting the prevalence of a specific health state by the corresponding well-being weight as:

2 $${WAHE}_{x}=\frac{1}{{l}_{x}}\sum_{k=1}^{n}\sum_{a=x}^{\omega }{\theta }_{k,a}{h}_{k,a}{L}_{a}$$

where WAHE_x_ is the well-being adjusted health expectancy at age $x$, ${l}_{x}$ is the number of life table survivors at age $x$, ${L}_{a}$ is the number of years lived at age $a,{h}_{k,a}$ is the share of the population in health state $k$ at age $a$, and ${\theta }_{k,a}$ is the health-related well-being weight assigned to health state $k$ at age $a$ with the weight for full health being ${\theta }_{k,a}$ = 1. The values for ${l}_{x}$ and *L*_*a*_ stem from a period life table, while the values ${h}_{k,a}$ and ${\theta }_{k,a}$ are derived from survey data. Note that the well-being weight ${\theta }_{k,a}$ corresponds to the health-related weight ${\pi }_{k,a}$ in Formula (1). We use a different parameter label in the WAHE formula to clarify that the weighting function in our indicator does not directly reflect the severity of the health state, but the extent of well-being associated with the health state. The derivation of the well-being weights is explained in more detail in the subsequent section.

WAHE can also be estimated with the double-decrement life table method or multistate life table method. The difference to the above formula is that, instead of being based on the prevalence of decreased health, life years lived in different health states are estimated based on transition probabilities between the states. Similarly, based on the transition probabilities between the states, WAHE can be estimated with multistate absorbing Markov chain models with rewards, as proposed first for the study of healthy longevity by Caswell and Zarulli (2018) and further specified for health transition probabilities from a panel data by Caswell and van Daalen (2021). In this case, the well-being weights would be entered directly into the reward matrix, analogous to weights for the severity of the disease in the estimations in Caswell and Zarulli (2018, p. 7).

### Well-Being Weights

Two technical issues must be addressed when estimating the weights for HALE: (1) whose values should be taken into account, i.e., the general population or only those who experience or experienced a particular disease or health problem, and (2) how those values should be collected (Brazier et al., 1999; Helgelsson et al., 2020; Richardson, 2002).

The first issue relates to two types of utility in microeconomics: decision utility and experienced utility. In QALY, used in health economics cost-benefit evaluations of health interventions, health state valuations are typically derived from general population surveys and are therefore based on decision utility, which is the predominant type of utility applied in microeconomics. The rationale for using this approach in health economics is that the population is seen as a collective of taxpayers who finance the health programs that are evaluated, and therefore, this population has the right to decide which health system investments should be prioritised (Helgelsson et al., 2020; Mukuria & Brazier, 2013; Richardson, 2002). This approach to collecting health state valuations from the general population is often criticised because most of its members have never experienced a specific health problem and thus cannot really asses to what extent this health problem might impact different aspects of their lives and well-being. Additionally, the general public has been shown to frequently overestimate the effect of a decreased health state on well-being. This is because they underestimate the extent to which they could adapt to this decreased health state (Dolan, 1996, 2007; Dolan & Green, 1998; Dolan & Kahneman, 2008; Helgelsson et al., 2020; Menzel et al., 2002; Mukuria & Brazier, 2013). An alternative approach of decision utility that has also been used to determine the disability weights for DALE and other indicators of the GBD is to base the health state valuation on assessments from a panel of health care professionals. Likewise, this approach has been criticised for not reflecting "individuals' differential ability to cope with their functional limitation" (Anand & Hanson, 1997, p. 689). An additional criticism of DALE weights is that since they are independent of the social context in which disabilities and health limitations occur, they do not accurately reflect the real impact of diseases on an individual's life (Anand & Hanson, 1998). While the GBD weights used for DALE and other measures have not been collected from health professionals since 2010 and instead come from population surveys (Salomon et al., 2015), the above-mentioned concerns raised by Anand and Hanson (1997, 1998) still hold.

The second approach for health states valuation relates to Kahneman's concept of "experienced utility" in economics (1994), i.e., information is collected by interviewing individuals who have experienced a specific state of health. The experienced utility was first proposed as a new standard in health policy evaluations by Ferrer-i-Carbonell and van Praag (2002) with data from a social survey, and later by Kahneman and Sugden (2005) and Brazier et al. (2005) with data from clinical studies.

To date, there is no universally accepted methodological approach for collecting information for health states valuation in surveys. The most frequently used methods are visual analogue scaling, standard gamble, time trade-off, and willingness-to-pay (Brazier et al., 1999; Essink-Bot & Bonsel, 2002; Feeny, 2002). As multi-attribute health-state classification systems describe more states than can be scored in surveys, valuation functions are often estimated from models based on a set of values obtained from a selection of states. These solutions are also criticised by the experienced utility approach described above, what led to the development of subjective well-being valuation (SWV). In the SWV approach, information on well-being associated with different health states comes directly from respondents who simultaneously assess their health across the study dimensions. According to Dolan and Metcalfe (2012) and Dolan et al. (2012), it is preferable to measure subjective well-being from answers to the question, "Overall, how satisfied are you with your life?". Responses to this question have been labelled in different ways, e.g., "happiness", "general satisfaction", and "subjective well-being" (Ferrer‐i‐Carbonell and Frijters, 2004) or "evaluative well-being" (Steptoe, 2019).

To our knowledge, the first application of SWV in health economics was by Ferrer-i Carbonell and Van Praag (2002), who propose using a method to estimate the valuation function by translating a decrease in subjective well-being caused by a decline in health into monetary terms. This technique estimates the compensating income for the loss in a health state, which refers to the additional income needed to return an individual to its state of well-being from before a given health loss. As this approach assumes cardinality of well-being, the weights are estimated with ordinary least squares regressions. Generally speaking, methods used in SWV either assume the cardinality or only ordinality of the well-being scale (MacKerron, 2012). However, Ferrer‐i‐Carbonell and Frijters (2004) demonstrated that assumptions of the cardinality or ordinality of answers concerning the well-being question do not affect the results in health studies. With the minimum assumption of the dependent variable being of ordinal character, we propose to estimate the health-related well-being weights for WAHE with the ordered probit model. Ordered probit models have been previously used by Cutler et al. (1997), Cutler and Richardson (1998), and Groot (2000) to estimate QALY weights to study population health in the USA, with the health state measured by chronic diseases and weights estimated based on self-rated health. WAHE weights are estimated as standardised coefficients from the model following the standardisation proposed by Cutler et al. (1997), where a corresponding coefficient is divided by the difference between the thresholds for excellent and poor health. A full description of the model and a discussion concerning other standardisation methods to derive the weights can be found in Online Resource 1. Analogous to the studies of Cutler et al. (1997), Cutler and Richardson (1998), and Groot (2000), the model is estimated separately for the subgroups of the population according to characteristics that are known to influence both health status and its effect on well-being. This is the context in which the decreased health occurs, e.g., country of residence, but also individual characteristics, such as sex and age (Di Lego et al., 2020; Groot, 2000; Jürges, 2007; Jylhä et al., 1998; Oksuzyan et al., 2019; Ulloa et al., 2013).

According to the SWV approach described above, the proposed WAHE indicator quantifies the overall burden of decreased health using health-related well-being based on information collected directly from those who experience specific health limitations. Like the health-related quality of life utility scores from QALYs (Drummond et al., 2015; Gold et al., 2002; Prieto & Sacristán, 2003; Whitehead & Ali, 2010), WAHE weights are expressed in terms of equivalent years of full health. Hence, a weight for a decreased health status in WAHE is expressed in terms of its equivalency in well-being to that of full health or, using Kaplan and Bush's (1982) terminology, in relation to a "Well-Year". A Well-Year is "the equivalent of a year of completely well life, or a year free of dysfunction, symptoms, and health-related problems" (p. 64). For example, the reduction in quality of life by half because of decreased health "will take away 0.5000 Well-Years over the course of one year" (Ibidem, p. 64) and hence result in this health state being assigned a weight of ${\theta }_{k,a}$ = 0.5.

## Validation of the WAHE Indicator

Validation of the proposed WAHE indicator is structured according to the guidelines of a Committee on Summary Measures of Population Health (Gold & Field, 1998). These include the following aspects (see Gold & Field, 1998, p. 14):

- “Reliability or reproducibility: a measure is reliable if repeated use under identical circumstances by the same or different users produces the same result;
- Validity: a measure is valid if it measures the properties, qualities, or characteristics it is intended to measure;
- Sensitivity or responsiveness: a measure is sensitive/responsive if it can detect differences or changes in population characteristics that interest users of the measure;
- Acceptability: a measure is acceptable if its intended users (and the constituencies upon which they depend) find the results of its application (e.g. a summary statistic) understandable, credible, and useful for their purposes;
- Feasibility or burdensomeness: a measure is feasible if users can collect the necessary data and perform the required analyses without imposing excessive administrative, economic, or other burdens on those whose participation or cooperation is needed;
- Universality or flexibility: a measure is universal/flexible if adaptable to the variability of problems, populations, settings, or purposes that face potential users;
- Documentation: a measure is documented when the methods, criteria, assumptions, and data employed in deriving or calculating the measure are clearly identified and publicly available.”

WAHE's validity, reliability, and sensitivity against different specifications of health, are additionally assessed in an empirical application.

### Theoretical Validation

**Reproducibility and Documentation:** A detailed documentation of the WAHE's estimation is provided along an R-code in an online repository at https://zenodo.org/record/5949229

**Validity:** WAHE is intended to be a SMPH as it combines information on mortality and health into a single number. WAHE is a SMPH of descriptive use, which means that it aims to describe differences in the distribution of health between populations, subgroups of populations, or changes in population health over calendar time (Essink-Bot & Bonsel, 2002; Murray et al., 2000; Van der Maas, 2002).

**Universality/Flexibility:** WAHE is a universal descriptive measure of population health as it can be applied to many dimensions of health and is characterised by minimum data requirements. The health status can be either self-assessed or measured by an objective health indicator. Due to flexibility in estimating life years, mortality data can come from registered data or death counts from a panel survey.

**Acceptability:** WAHE is constructed according to the standard formula for HALE. The element of WAHE, which is innovative for a measure of descriptive use, is the subjective well-being valuation of health states. This valuation method has already been applied in the estimation of QALY in health economics cost-benefit evaluation studies (Dolan, 2011; Ferrer‐i‐Carbonell & Van Praag, 2002; Mukuria & Brazer, 2013). Contrary to standard QALY valuations and valuations in DALE, weights used in the WAHE indicator are transparent and estimated exclusively based on observed data, not on theoretical models, as is the case with other valuation functions.

**Feasibility/Burdensomeness:** When WAHE is estimated based on subjective self-assessed health status from survey data, its data requirements are minimal since many surveys include both information on health status and well-being of respondents. Contrary to that, weights used in DALE and QALY require additional representative surveys or expert assessments to derive the valuations of health states.

**Sensitivity/Responsiveness:** WAHE is based directly on health states' prevalence and mortality rates or transition rates between health states. Its aim is to monitor changes or differences in these statistics.

### Empirical Validation of the WAHE Indicator

The validity and reliability of WAHE to quantify population health are assessed by comparing its estimates for 29 European countries with those of other commonly used SMPHs: LE, HE, and DALE. As health is a complex phenomenon, no single concept or quantification of population health could be used as a reference for the new indicator. Each of the indices that WAHE is validated against quantifies a different population health dimension. This implies that a full empirical assessment of the new indicator requires its comparisons with all other measures and a comprehensive study where the other measures are contrasted against each other.

#### Methods of Empirical Validation

First, we study the degree to which rankings of countries according to the values of WAHE and other SMPHs coincide with a Spearman correlation. Next, to assess WAHE's validity and reliability as a measurement instrument of population health, we study its agreement with the other SMPHs and reliability to quantify population health. We follow the guidelines of quality diagnostics of newly developed classifications, scales, and other measurement instruments in clinical studies (Kottner et al., 2011). An agreement stands for comparability between results obtained by pairs of SMPHs in the study group of countries. We assess agreement between SMPHs graphically by Bland–Altman plots. Two measurements are in agreement if the difference in their values lies within the limits of agreement (LoA). In case of no linear relationship between the mean values of the two indices and their difference, these limits are estimated based on the mean and 1.96 standard deviation of the differences between the measurements. In the case of the SMPHs in this empirical example, we observe a significant linear relationship between the mean values of the two indices and their difference, and hence LoA are estimated based on the residual values of the linear model (Bland & Altman, 1999).

The third studied feature, reliability, indicates to what extent a measurement instrument can differentiate different levels of the study phenomenon (Kottner et al., 2011). In the particular case of SMPH the question of reliability needs to be framed differently, i.e., whether this group of indicators can differentiate between population health across the study countries and whether the inclusion of WAHE increases the group's reliability. High reliability signifies that the sum of systematic differences in health levels between countries constitutes a high share of the sample's total variability. This also implies that in the situation of high reliability, the measurement error, which is the sum of differences between values obtained for the indices for each country, is small. Following the guidelines by Kottner et al. (2011) for estimating and reporting reliability and agreement in epidemiological studies, the intraclass correlation coefficient (ICC) is applied in this study as the preferred statistical method to assess reliability for continuous variables such as any SMPH. First, we assess the reliability of the whole group of SMPHs to quantify population health across countries. Next, we study the change in the group reliability when single indicators are excluded from the group. In particular, we assess reliability using a two-way mixed-effects consistency model (Koo & Li, 2016; McGraw & Wong, 1996; Shrout & Fleiss, 1979). A detailed description of the methods used to assess agreement and reliability is provided in Online Resource 1.

#### Data and Methods to Estimate WAHE

WAHE is estimated with health specified initially in one of the dimensions of the three Minimum European Health Module (MEHM): chronic morbidity captured by the question "Do you have any longstanding illness or health problem?" with the answer options "Yes" and "No", limitations in activities of daily living with the Global Activity Limitation Indicator (GALI), based on the question: "For at least the past six months, to what extent have you been limited because of a health problem in activities people usually do? Would you say you have been…?" with the answer options "Severely limited", "Limited but not severely" and "Not limited at all", and self-rated health (SRH), based on the question "How would you rate your health in general?" using a scale ranging from 1 ("excellent") to 5 ("poor"), and then simultaneously in the three health dimensions. We define full health as having no chronic morbidity and/or being not limited at all and/or excellent general health, respectively. Although these dimensions are strongly correlated at the individual level, none has been shown to be a fully comparable objective measure of health across countries and different socio-economic groups (Au & Johnston, 2014; Berger et al., 2015; Lazarevič & Brandt, 2020). Hence, they reflect different dimensions of health and are also differently influenced by contextual factors. For example, SRH is significantly affected by cross-country differences in health perceptions (Jürges, 2007; Jylhä et al., 1998). The effect of morbidity and disability on the limitations represented in GALI is affected by the availability of publicly financed facilities and services for those with health limitations (Anand & Hanson, 1998). Chronic morbidity reporting depends on respondents' knowledge, which is influenced by health insurance systems, health utilisation, and medical technology levels (Saito et al., 2014). Well-being is assessed on answers to the question "Overall, how satisfied are you with your life?" with options ranging from 1 ("not satisfied") to 10 ("completely satisfied").

The health status and well-being data are taken from the EU Statistics on Income and Living Conditions Survey in 2018 (Eurostat, 2021b). The life tables and LE for 2018 required to estimate WAHE and HE come from Eurostat (2022). When estimating the population share in selected health states, we apply cross-sectional weights from the EU-SILC. The DALE estimates are taken from GBD (2020). Respondents from the 29 countries comprising the EU-SILC cross-sectional 2018 dataset were included in this study. Germany and Malta were excluded because of different age coverage. Tables A1 and A2 in Online Resource 2 present the distribution of respondents per country and the number of respondents included in the separate analyses for each of the health dimensions and for estimating the well-being weights. Since no imputation method was applied for missing data and observations with missing values for a state across a studied health dimension are removed, there are small differences in the sample size for each analysis. EU-SILC population data is available for all study countries for single ages between 17 and 79 years and the open age interval 80+. The SMPHs have been estimated at age 15 to make them comparable with DALE estimates available for this age. Like Eurostat (2021a), we assume that the prevalence of decreased health at ages 15 and 16 is equal to that at age 17. The prevalence of health states across the study dimensions was estimated for age groups 15–29, 30–39, 40–49, in five-year age groups between ages 50 and 79, and open-ended for 80+ years. Years lived in different health states are derived with the Sullivan method.

**Table 1:** Mean values of health-related well-being weights and mean age-standardised prevalence of health states in 29 European countries by sex, 2018. *Source*: Authors' estimations based on Eurostat (2021b)

Health dimension	Weight		Prevalence	
	Men	Women	Men	Women
*Chronic morbidity*				
No	1.00	1.00	0.69	0.58
Yes	0.87	0.86	0.39	0.42
*Activity limitations*				
Not limited	1.00	1.00	0.68	0.64
Limited	0.87	0.87	0.22	0.25
Severely limited	0.72	0.73	0.10	0.11
*Self-rated health*				
Excellent	1.00	1.00	0.23	0.20
Very good	0.89	0.89	0.39	0.38
Good	0.78	0.77	0.26	0.27
Fair	0.63	0.63	0.10	0.12
Poor	0.51	0.50	0.02	0.03
*Multiple (chronic morbidity, activity limitations, self-rated health)*				
No, not, excellent	1.00	1.00	0.21	0.19
No, not, very good	0.89	0.88	0.28	0.27
No, not, good	0.79	0.79	0.06	0.06
Yes, not, very good	0.91	0.91	0.05	0.05
Yes, moderately, good	0.77	0.76	0.02	0.03

Mean values of well-being weights across age groups and countries; health states in the multiple dimensions selected if their mean prevalence equals to at least 0.03; standard population is the total population of the 29 European countries

**Table 2:** Summary statistics for life expectancy (LE), disability-adjusted life expectancy (DALE), health expectancy (HE) and well-being adjusted health expectancy (WAHE) for three dimensions of health: chronic diseases (Chronic), activity limitations (GALI), self-rated health (SRH), and simultaneously across the three dimensions (Multi.) at age 15 in 29 European countries, 2018. *Source*: Authors' estimations based on Eurostat (2021b, 2022) and GBD (2020)

Statistic	LE	DALE	HE				WAHE			
			Chronic	GALI	SRH	Multi.	Chronic	GALI	SRH	Multi.
	*Men*									
Mean	62.7	54.0	43.7	48.0	17.7	16.5	60.3	60.3	54.9	54.7
Standard deviation	3.6	2.7	5.2	5.0	6.6	6.1	3.8	3.7	4.9	5.1
Variation coefficient (in %)	6	5	12	10	37	37	6	6	9	9
	*Women*									
Mean	68.3	56.1	43.7	48.2	16.5	15.3	64.9	64.9	58.6	58.2
Standard deviation	2.2	1.9	5.7	4.9	6.6	6.2	2.5	2.3	4.0	4.2
Variation coefficient (in %)	3	3	13	10	40	40	4	4	7	7

Variation coefficient V = SD/mean * 100%.

The well-being weights for the health states are derived based on ordered probit models estimated separately by country and sex. As both prevalence of decreased health and well-being depend on age, we included age and age squared as control variables in the model. Although the effect of decreased health on well-being is also likely to depend on age, an interaction effect between health state and age group has not been included in the models because of the small sample sizes of some of the health states and age interactions.

All estimations were carried out in R. We used the R packages *MASS* (Venables & Ripley, 2013) to estimate well-being weights and *psych* (Revelle, 2015) for the empirical index evaluation (agreement and reliability).

## Results

**Fig. 1:**
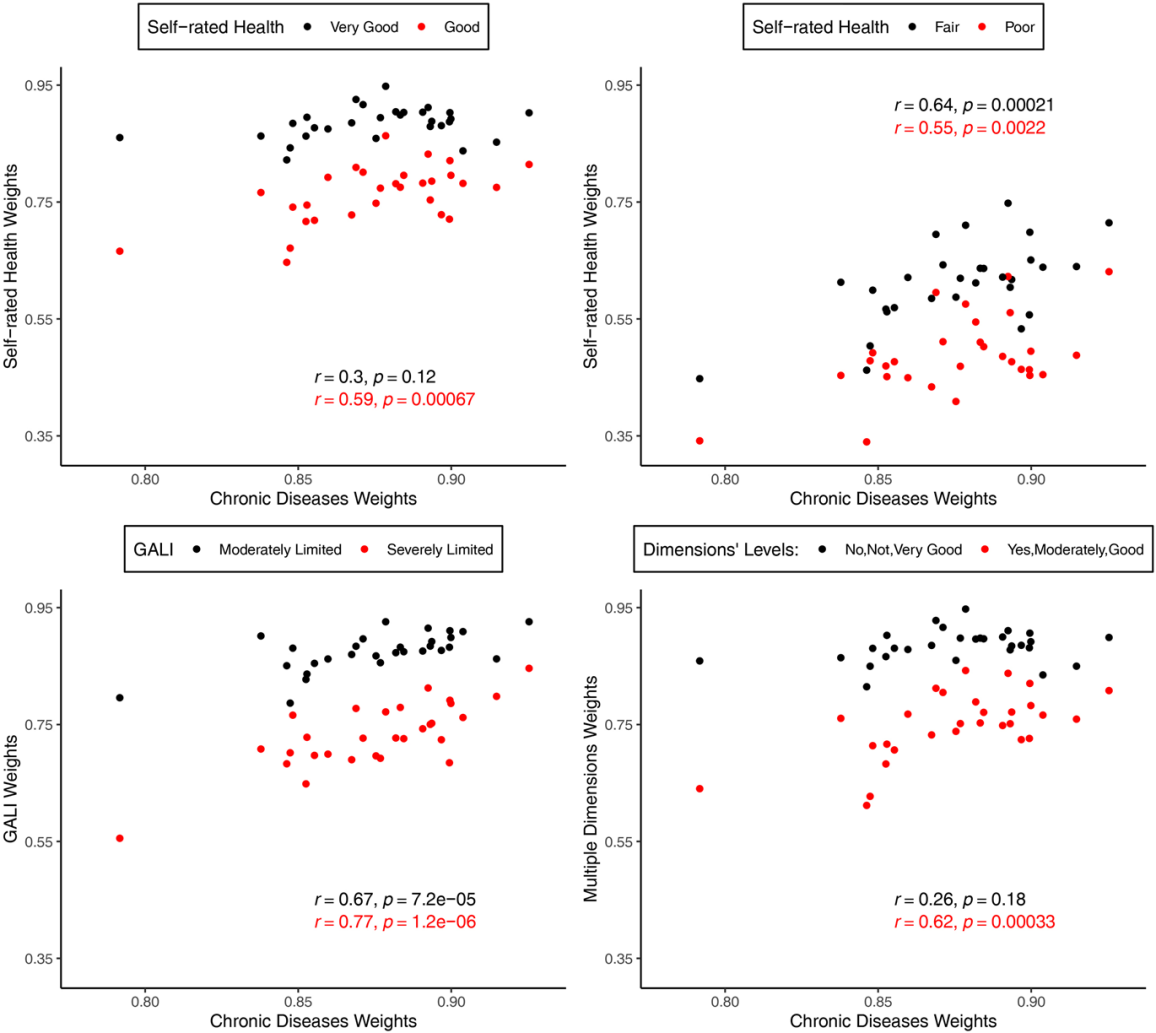
Well-being weights for health states in 29 European countries, 2018, both sexes. *Source*: Authors' estimations based on Eurostat (2021b)

Table 1 presents descriptive statistics of mean values of health-related well-being weights and mean age-standardised prevalence of health states for the total sample of the 29 European countries included in this empirical application. The standard population is the total population of all countries. Weights and health state prevalence values in single European countries are included in Online Resource 2 (Tables A3 and A4, Figures A1, A2). Given that health is recognised as one of the most important determinants of well-being (Graham, 2008; Steptoe, 2019; Steptoe et al., 2015; Wu et al., 2014), it is no surprise that the well-being weights for a year lived in decreased health fall below one and that the weights decrease with the severity of the health limitations. For example, for SRH a year lived in very good health is equivalent in well-being to about 10.5 months (average well-being weight = 0.89) of full health defined as "excellent" health. For good SRH, this value decreases to 9.0 months (weight = 0.77). The most considerable effect that decreased health has on well-being is for fair and poor SRH: A year lived in fair SRH is equivalent on average to 7.5 months lived in “excellent” SRH (weight = 0.63), while a year of poor SRH is equivalent to only half a year in full health (weight = 0.50). Well-being weights for decreased health are also characterised by a positive and significant correlation across the study countries (Fig. 1), indicating the importance of the context in which the decreased health occurs for its effect on well-being and, as a consequence, for the construction of WAHE.

Table 2 presents summary statistics for the distribution of SMPHs across 29 European countries. Indices for individual countries are included in Online Resource 2 (Tables A5 and A6). The average male LE at age 15 in European countries was 63 years, of which 17 to 44 years were spent in full health (HE), 55 to 60 years were spent in well-being equivalent to full health (WAHE), and 54 years were spent disability-free (DALE). For women, the corresponding numbers are LE of 68 years, HE of 15 to 48 years, WAHE of 58 to 65 years, and DALE of 56 years. The lowest HE and WAHE values appear for measures based on SRH, regardless of whether the health state is specified across one or multiple dimensions. Additionally, there are considerable differences in HE between countries based on SRH: The coefficients of variation equal to 37 percent for men and 40 percent for women. The low HE based on SRH and its substantial variation across the study countries result from the fact that full health is defined by only a single level, which is the highest possible level of “excellent” SRH, and the variation of the prevalence of this single health state across countries is large (see also Figures A1 and A2 in Online Resource 2). This result demonstrates that as being based on a subjective threshold for full and decreased health, HE is not reliable as an indicator of population health (see also Murray et al., 2002). As shown by Jürges (2007) and acknowledged in Eurostat's accompanying technical guidelines for official HE estimates (2021a), the wide variation in the prevalence of any specific level of SRH in social surveys arises from the substantial cultural differences in the comprehension and interpretation of the health rating scales. The WAHE indicator overcomes this issue because it avoids relying on a single threshold between full and decreased health. Instead, WAHE simultaneously accounts for all SRH levels.

The country rankings according to the four WAHE measures are highly correlated, as indicated by the Spearman correlation coefficients above 0.9 (Table 3). In particular, country rankings for WAHE based on SRH and multiple dimensions are almost identical, with a correlation coefficient close to one. Additionally, WAHEs for the different health dimensions are strongly correlated with LE and DALE. However, it should be noted that country rankings according to DALE and LE are also nearly identical. These similarities between the rankings of countries according to LE, DALE, and WAHE are a consequence of the weighting of multiple health states in the HALE formula, which is used for both WAHE and DALE. The HALE formula applies weights to the prevalence of decreased health, and in empirical studies, weights close to one are applied to health states with the highest prevalence (e.g. good SRH in WAHE), while only rare health states (e.g. poor SRH) have low weights. Consequently, decreased population health leads to only a small decrease in the total number of life years and, therefore, the health-adjusted indicators WAHE and DALE remain close to the value of LE. The most striking result is that the country rankings according to DALE are not significantly correlated to rankings according to any of the HE measures. On the other hand, this is not the case for the WAHE indicators. The correlation coefficients are statistically significant for most pairs of WAHEs and HEs, with chronic morbidity among men being the only exception.

**Table 3:**
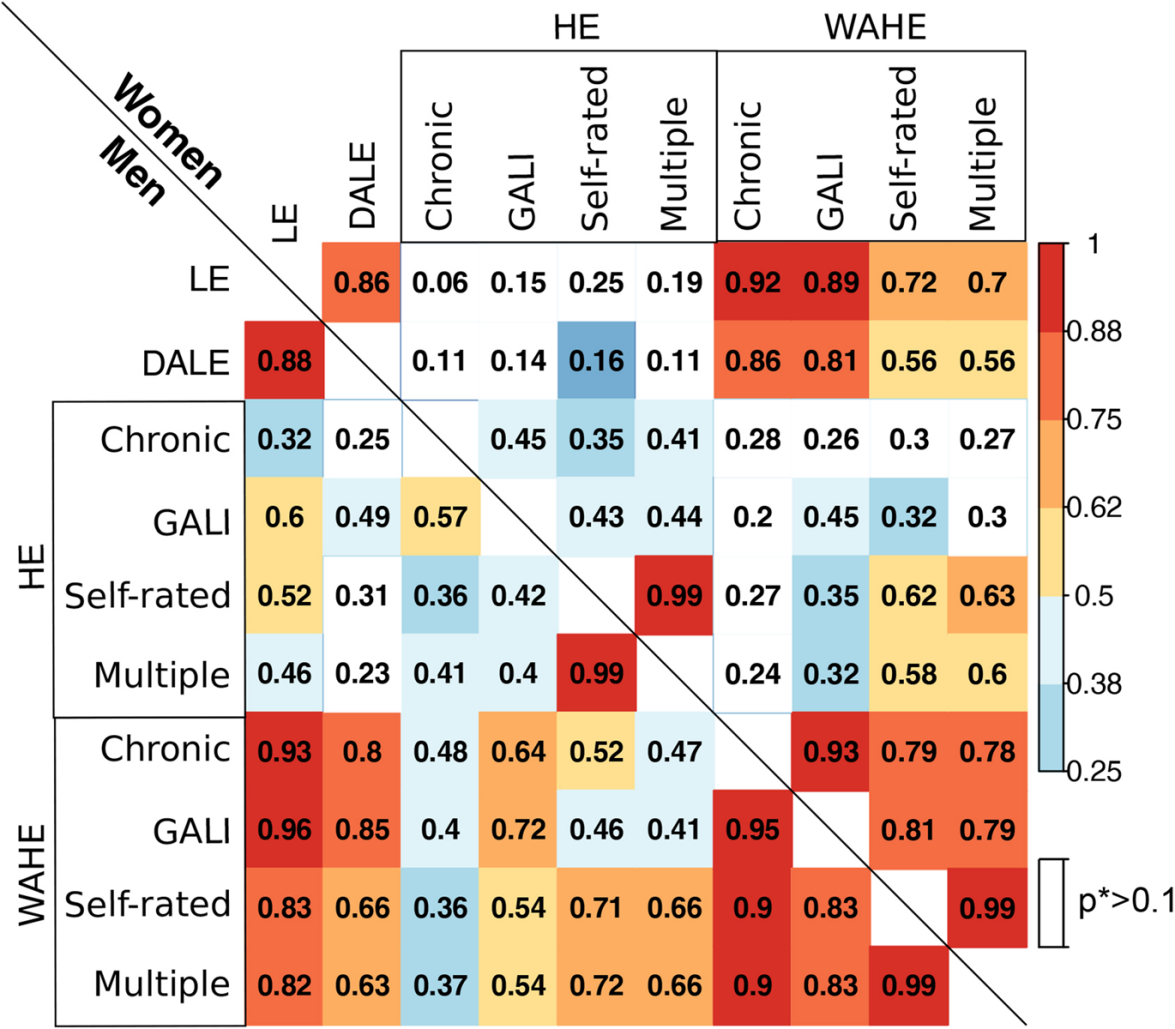
Spearman correlation coefficients for the summary measures of population health at age 15 in 29 European countries, 2018. *Source*: Authors' estimations based on Eurostat (2021b, 2022) and GBD (2020)

Notes: LE = life expectancy; DALE = disability-adjusted life expectancy; HE = health expectancy; WAHE = well-being adjusted health expectancy; three dimensions of health: chronic diseases (Chronic), activity limitations (GALI), self-rated health (Self-rated), and simultaneously across the three dimensions (Multiple); White squares represent non-significant correlation at *p > 0.1

**Fig. 2:**
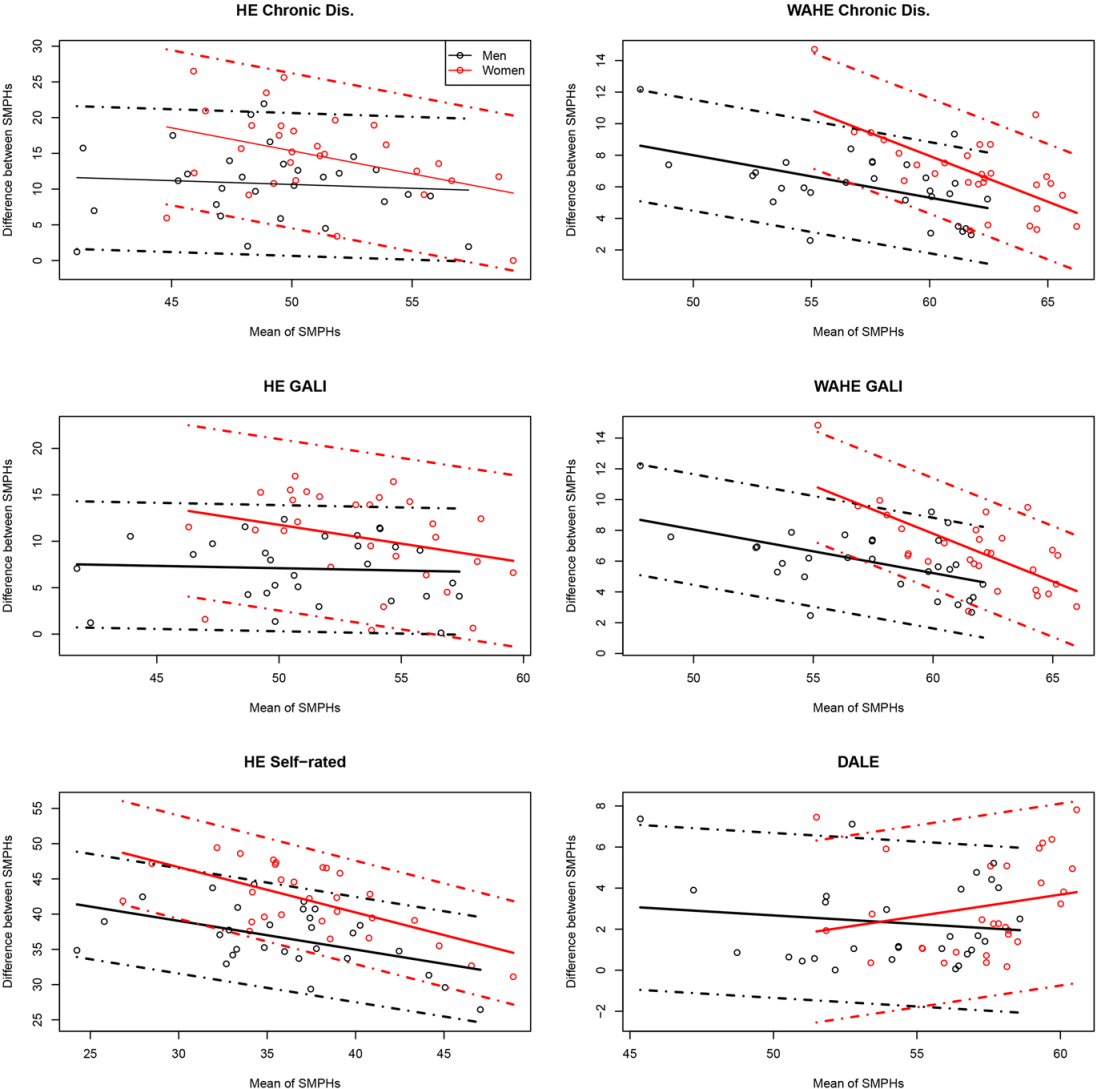
Bland-Altman plots for agreement between WAHE based on self-rated health and other summary measures of population health at age 15 in 29 countries by sex, 2018. *Note*: Dots represent country observations; solid lines represent the linear model; dashed lines represent the 95% confidence intervals. *Source*: Authors' estimations based on Eurostat (2021b, 2022) and GBD (2020).

**Fig. 3:**
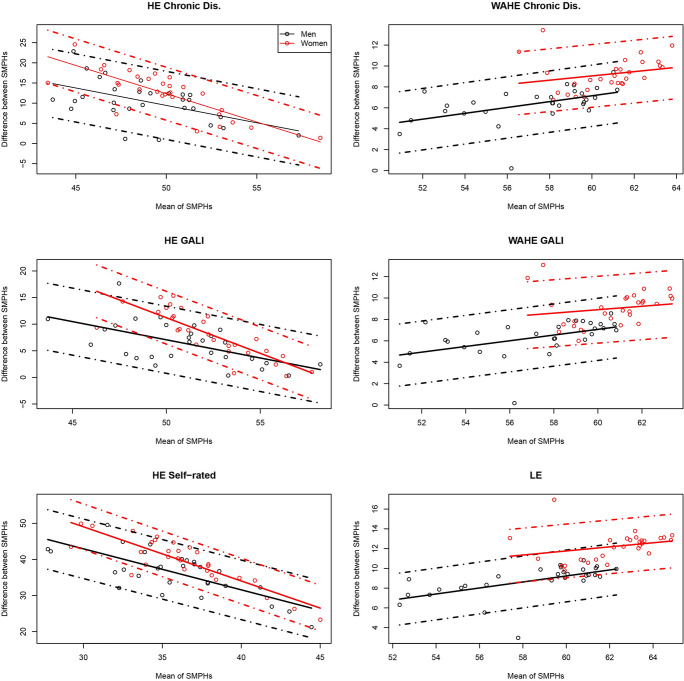
Bland-Altman plots for agreement between DALE and other summary measures of population health at age 15 in 29 countries by sex, 2018. *Note*: DALE = disability-adjusted life expectancy; dots represent country observations; solid lines represent the linear model; dashed lines represent the 95% confidence intervals. *Source*: Authors' estimations based on Eurostat (2021b, 2022) and GBD (2020).

Figure 2 shows Bland–Altman plots for the agreement between WAHE based on SRH and the other SMPHs. Online Resource 2 Figures A3 and A4 depict nearly identical Bland–Altman plots for WAHE based on chronic morbidity and GALI. Figure 3 shows Bland–Altman plots of the agreement between DALE and the remaining indices, which demonstrates agreement between the pairwise indicators: With very few exceptions, the measurements are closer to each other in the Bland–Altman plots than the 95 percent limits of agreement. The limits of agreement in the Bland–Altman plots are wide and range between 2 and 10 years. The widest limits of agreement, which indicate the largest absolute differences between indicator values, are between HE based on chronic morbidity and WAHEs, independent of the health dimension. However, these large absolute differences do not signify a large measurement error in the SMPH. Instead, they occur because health is a complex multidimensional phenomenon, and the indicators quantify these dimensions differently.

A further interesting feature revealed by the Bland–Altman agreement plots is the linear relationship between the mean value of the pairs of indicator values and their absolute differences. Aside from the pairwise comparisons of WAHEs and DALE, each linear relationship between WAHEs and the remaining SMPH is negative. Combined with the positive correlation between the measures described above, this means that, as the values of both indicators increase, the absolute differences between them decrease. In other words, the indicators report similar values in countries with a higher level of population health and diverge in countries with a lower level of population health. The difference between two SMPHs relative to their mean value can also be seen as an indicator of how precisely we capture the population health level based on these measures. Hence, our result implies that the higher the population health level, the more precisely we can measure it with any of the SMPHs (except the pairs of measures that include DALE). The opposite is also true: At lower levels, the differences in the estimates of population health between the two measures are greater. An alternative explanation could be that populations with higher LE also have a better overall health status, regardless of how health is measured. Interestingly, the slope of the fitted linear models for all pairs of indices is higher for women than for men. The higher slopes among women result from larger differences between indices at their low values, while the gap between measures at high index values is similar for both sexes. However, our empirical findings show that the opposite is true for the relationships between DALE and WAHE: as the indicators' values increase, their differences become larger, especially for women.

Table 4 shows the intraclass correlation coefficients for all studied SMPHs combined and the same group with single indicators omitted for the 29 included European populations. The group of SMPHs is characterised by its high reliability for measuring population health across European countries. This reliability is higher for men, where the intraclass correlation coefficient equals 0.92, compared to 0.86 for women. Only minor changes occurred in the measurements' reliability when a single measure was added to the group. For most measures, their inclusion in the group of indices does not change the group's reliability or increases it only marginally. For both sexes, however, excluding HE based on chronic morbidity increases the reliability of this group of measures. The same effect is observed for women in the case of DALE. Both effects were only minor, however. Nevertheless, the small sample size led to wide confidence intervals in the correlation coefficients (Streiner et al., 2015), making these changes to reliability statistically insignificant.

**Table 4:** Intraclass correlation coefficients (ICC) for all summary measures of population health and after excluding single measures in 29 European countries, 2018. *Source*: Authors' estimations based on Eurostat (2021b, 2022) and GBD (2020)

Statistic	All	Excluding								
		DALE	HE				WAHE			
			Chronic	GALI	SRH	Multi.	Chronic	GALI	SRH	Multi.
Men	0.92	0.92	0.93	0.92	0.92	0.92	0.91	0.91	0.90	0.90
Women	0.86	0.87	0.88	0.86	0.83	0.83	0.85	0.85	0.83	0.83

LE = life expectancy; DALE = disability-adjusted life expectancy; HE = health expectancy; WAHE = well-being adjusted health expectancy; three dimensions of health: chronic diseases (Chronic), activity limitations (GALI), self-rated health (SRH), and simultaneously across the three dimensions (Multi.)

## Summary and Discussion

We propose a new summary measure of population health, which aims at describing differences in the distribution of health between populations, subgroups of populations, or changes in population health over calendar time. It combines information on mortality, health, and health-related well-being. The well-being adjusted health expectancy (WAHE) belongs to the family of HALE indicators. The weights applied to years lived in different health states quantify the well-being associated with a given health state compared to the well-being in full health. Analogous to DALE from the GBD, WAHE measures the expected health-related equivalent length of life. Therefore, WAHE is an explicit health indicator and must be distinguished from the recently proposed indicator “Years of Good Life” (YoGL) which was developed as an alternative indicator of well-being and contains physical and mental health as elements together with the other two domains “out of poverty” and “positive life satisfaction” (Lutz et al. 2021).

We derive WAHEs for the three health indicators of the Minimum European Health Module—SRH, GALI, and chronic health problems—and validate the proposed indicator theoretically and empirically according to the guidelines of a Committee on Summary Measures of Population Health (Gold & Field, 1998). Therefore, we discuss the validity, universality, feasibility, and sensitivity of the WAHE indicator. We assure reproducibility of the measure by providing detailed documentation and the computer code in an online repository. In the empirical part of the study, WAHE is estimated for 29 European countries based on the 2018 EU-SILC data. We assess rank correlation, agreement, and reliability of WAHE against other commonly used SMPHs, i.e., LE, HE, and DALE.

In addition to its theoretical strengths, the empirical component of the study confirms that WAHE is a useful SMPH. The ranking of population health across the study countries according to WAHE is similar to the other commonly used SMPHs. WAHE performs well, independent of the health dimensions applied to specify health, as demonstrated by its agreement with all other considered SMPHs and the high reliability of this group of indices. The ranking of countries according to WAHE is also highly positively correlated with the ranking based on LE and DALE. The correlation between rankings of countries between HE and WAHE is also positive and mostly significant, and between HE and DALE is mostly non-significant. The high correlation of country rankings according to WAHE and DALE with LE—as well as the general closeness of their values—results from the fact that most of life years are spent in a health state equivalent to full health. The more pessimistic picture painted by HE is due to its dichotomous nature: Life years can be spent either in good or poor health, and all life years spent in a less than full health state do not count at all for HE. Moreover, the most prevalent, relatively good health states have high weights in WAHE and DALE, while the low weights are assigned to states of higher severity of health decrease but also low prevalence. This implies that the most prevalent states of decreased health, which receive a zero weight in HE and hence lower most the number of years lived in good health in this indicator, are weighted in WAHE and DALE with a weight close to one, which otherwise is the weight of full health, making the values of the two indicators closer to each other and to LE. This implies that the high correlation of country rankings according to LE, WAHE, and DALE and the closeness of their values do not necessarily point at a redundancy of the information on population health of the three indicators.

Compared to DALE, WAHE is easier and cheaper to obtain and replicate. WAHE is based on easily accessible social survey data and has a straightforward valuation function, which accounts for the consequences of decreased health among those who have experienced it. Moreover, WAHE accounts for the fact that decreased health occurs in a social context, which shapes differences in the prevalence of specific health conditions and their consequences on people's well-being. DALE does not differentiate disability weights between countries. Using the extreme example of HE where full health was defined as excellent SRH, we also demonstrated that WAHE describes a population's average health state in a much more comprehensive manner than HE as it summarises information on population health without being driven by the subjective dual distinction between full and decreased health. To conclude, although there is no simple answer to which of the SMPHs is the best measure for analysing population health across countries and between subpopulations, WAHE is a useful indicator of population health. It performs at least as well as DALE and HE, but overcomes their best-recognised limitations.

### Supplementary Information

Below is the link to the electronic supplementary material.

Supplementary file1 (PDF 1642 KB)

Supplementary file1 (PDF 1152 KB)

## Data Availability

The datasets analysed during the current study are publicly available from Eurostat, EU Statistics on Income and Living Conditions survey for the year 2018 (EU-SILC 2018) upon institutional access application and the public domain of the Eurostat available at https://appsso.eurostat.ec.europa.eu/nui/show.do?dataset=demo_mlifetable&lang=en. The responsibility for all conclusions drawn from the data lies entirely with the authors.

## Footnotes

1 Note that the term DALE was replaced by the term HALE (“health-adjusted life expectancy”) by WHO in 2001 (see, Hyder et al., 2012). Nonetheless, we refer to the GBD indicator as DALE in this paper because we use the term HALE for the general concept and formula of adjusting a population's life expectancy (LE) by the population's health status. Unfortunately, there is no agreement in the literature about such a general term. Moreover, also the terms used for specific SMPH indicators vary between publications what complicates the incorporation of different indicators and concepts. Therefore, the consistent use of terms and definitions in this paper necessitated the aforementioned compromise to use for the term HALE for the general measurement concept and the term DALE for the concrete GBD indicator.

## Code Availability

Code in R is available at https://zenodo.org/record/5949229.
