# Correction: Well-Being Adjusted Health Expectancy: A New Summary Measure of Population Health

**DOI:** 10.1007/s10680-023-09648-5

**Published:** 2023-02-28

**Authors:** Magdalena Muszyńska-Spielauer, Marc Luy

**Affiliations:** Vienna Institute of Demography (OeAW), Wittgenstein Centre for Demography and Global Human Capital (IIASA, OeAW, University of Vienna), Vienna, Austria

**Correction: European Journal of Population (2022) 38:1009–1031** 10.1007/s10680-022-09628-1

The original online version of this article was revised: In this article, the statement in the Funding information section was incorrectly given as Open Access funding provided by the Österreichische Akademie der Wissenschaften; it should have read “European Research Council”.

