# Supplementary material for: Well-Being Adjusted Health Expectancy: A New Summary Measure of Population Health
Source: Eur J Popul. 2022 Aug 8;38(5):1009–31. doi: 10.1007/s10680-022-09628-1 (PMC9726765; doi:10.1007/s10680-022-09628-1)
Supplement: Supplementary file 1 — Supplementary file1 (PDF 1642 KB) [file 10680_2022_9628_MOESM1_ESM.pdf]

Online Supplementary Material 1: Additional Tables and Figures

Table A1: Number of respondents in the 29 countries in EU-SILC 2018, number of respondents included in the analyses for each health dimension and in the estimation of the well-being weights, men.

Country	Total	Chronic Diseases	GALI	Self-rated Health	Multiple Health States	Well-being
Austria	4,991	4,986	4,988	4,989	4,984	4,511
Belgium	5,382	5,330	5,330	5,329	5,329	4,782
Bulgaria	7,012	7,008	7,008	7,008	7,008	5,120
Switzerland	6,007	5,144	5,141	4,839	4,829	4,839
Cyprus	4,283	4,280	4,280	4,280	4,280	3,701
Czech Rep.	7,495	4,122	4,122	4,122	4,122	4,116
Denmark	4,815	2,626	2,613	2,627	2,609	2,616
Estonia	5,717	4,160	4,159	4,156	4,155	4,162
Greece	23,480	23,480	23,480	23,480	23,480	22,000
Spain	13,536	13,434	13,434	13,434	13,434	13,263
Finland	9,739	5,050	5,046	4,705	4,702	4,712
France	9,475	9,151	9,166	9,149	9,134	6,118
Croatia	8,747	8,681	8,681	8,681	8,679	4,393
Hungary	6,387	6,369	6,353	6,376	6,342	5,252
Ireland	4,103	4,103	4,103	4,103	4,103	2,404
Italy	18,874	18,596	18,587	18,645	18,584	13,379
Lithuania	4,288	4,035	4,025	2,230	2,167	2,242
Luxembourg	4,161	4,133	4,134	4,134	4,128	2,661
Latvia	4,593	4,376	4,376	4,376	4,376	2,913
Netherlands	11,171	5,588	5,530	5,630	5,496	5,641
Norway	5,839	3,103	3,073	3,104	3,073	3,097
Poland	15,624	12,750	12,731	12,777	12,702	7,527
Portugal	13,687	13,663	13,664	13,639	13,638	7,841
Romania	7,437	7,437	7,437	7,437	7,437	6,411
Serbia	6,835	6,835	6,835	6,835	6,835	6,010
Sweden	5,724	2,901	2,888	2,858	2,838	2,846
Slovenia	10,738	4,076	4,076	4,076	4,076	3,424
Slovakia	6,278	6,178	6,168	6,247	6,121	6,278
UK	15,004	14,687	14,686	14,692	14,682	7,876
Total	251,422	216,282	216,114	213,958	213,343	170,135

Source: Authors' estimations based on Eurostat (2021c).

Table A2: Number of respondents in the 29 countries in EU-SILC 2018, number of respondents included in the analyses for each health dimension and in the estimation of the well-being weights, women.

Country	Total	Chronic Diseases	GALI	Self-rated Health	Multiple Health States	Well-being
Austria	5,642	5,642	5,639	5,641	5,638	5,288
Belgium	5,700	5,650	5,650	5,650	5,649	5,162
Bulgaria	7,927	7,924	7,924	7,924	7,924	6,812
Switzerland	6,534	5,756	5,748	5,450	5,427	5,448
Cyprus	4,919	4,917	4,917	4,917	4,917	4,309
Czech Rep.	8,559	6,566	6,566	6,566	6,566	6,562
Denmark	5,134	2,927	2,918	2,922	2,911	2,917
Estonia	6,573	5,698	5,698	5,697	5,696	5,699
Greece	25,423	25,423	25,423	25,423	25,423	24,090
Spain	14,836	14,726	14,726	14,726	14,726	14,552
Finland	9,485	4,672	4,667	4,431	4,428	4,437
France	10,477	10,216	10,230	10,217	10,197	8,435
Croatia	9,625	9,536	9,535	9,535	9,534	6,297
Hungary	7,978	7,962	7,948	7,972	7,936	7,464
Ireland	4,387	4,387	4,387	4,387	4,387	3,125
Italy	21,095	20,735	20,736	20,798	20,720	16,167
Lithuania	5,467	5,271	5,265	4,116	4,039	4,131
Luxembourg	4,365	4,343	4,350	4,348	4,340	3,317
Latvia	6,192	6,044	6,044	6,044	6,044	5,155
Netherlands	11,942	6,776	6,682	6,837	6,628	6,852
Norway	5,793	2,836	2,799	2,836	2,798	2,822
Poland	17,756	15,945	15,901	15,971	15,873	13,299
Portugal	15,673	15,647	15,649	15,633	15,631	10,962
Romania	8,100	8,100	8,100	8,100	8,100	7,349
Serbia	7,152	7,152	7,152	7,152	7,152	6,474
Sweden	5,680	2,922	2,907	2,881	2,860	2,870
Slovenia	11,186	4,593	4,593	4,593	4,593	5,245
Slovakia	7,310	7,200	7,209	7,287	7,152	7,310
UK	16,365	16,140	16,136	16,138	16,130	9,331
Total	277,27 5	245,706	245,49 9	244,192	243,419	211,881

Source: Authors' estimations based on Eurostat (2021c).

Figure A1: Age standardized prevalence of health states across the study health dimensions in all 29 European countries (black points) and across the single countries (grey points) in 2018, men.

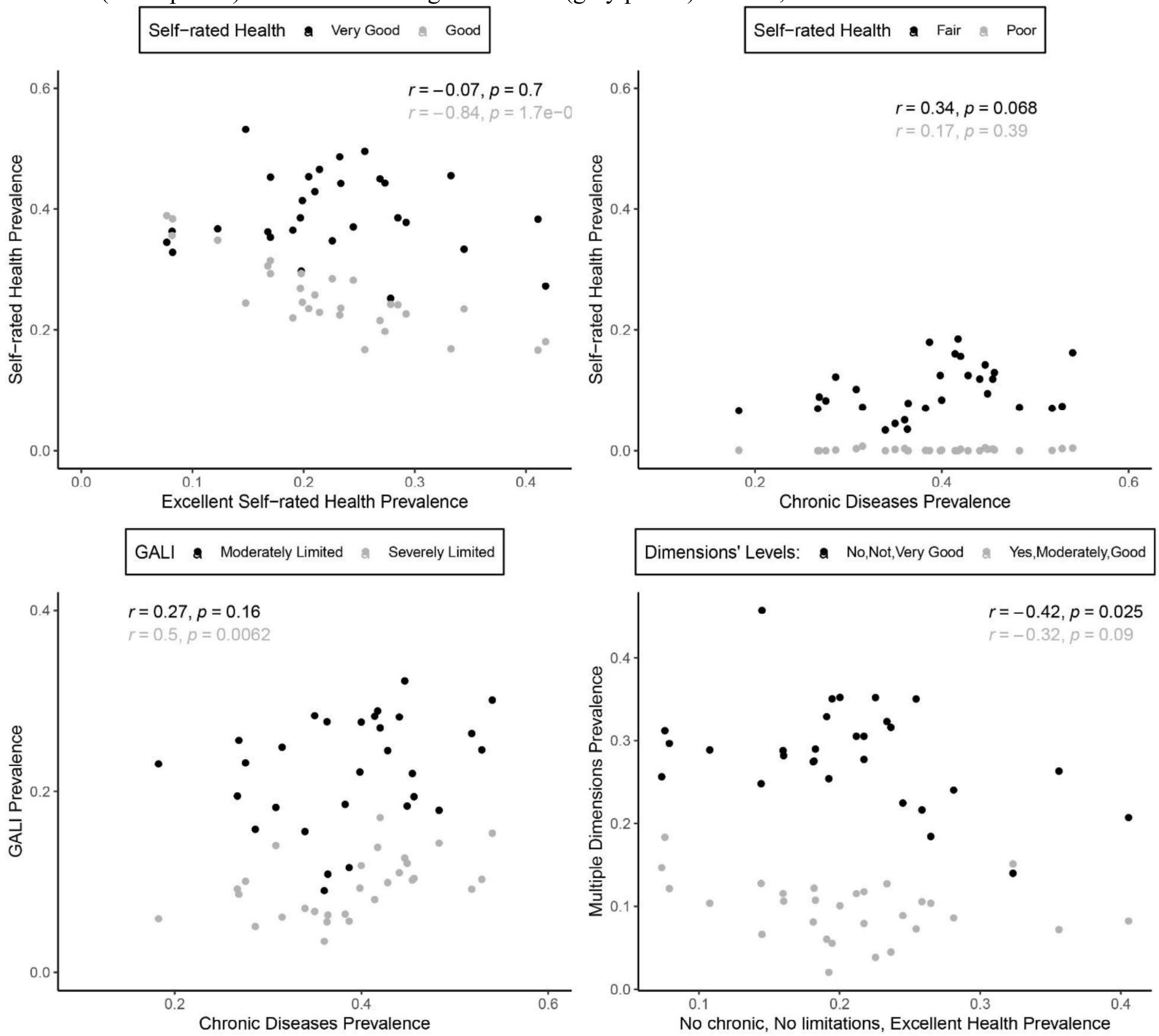

Notes: Standard Age= Eurostat for EU-28 population for 2018.
Source: Authors' estimations based on Eurostat (2021c).

Figure A2: Age standardized prevalence of health states across the study health dimensions in all 29 European countries (black points) and across the single countries (grey points) in 2018, women.

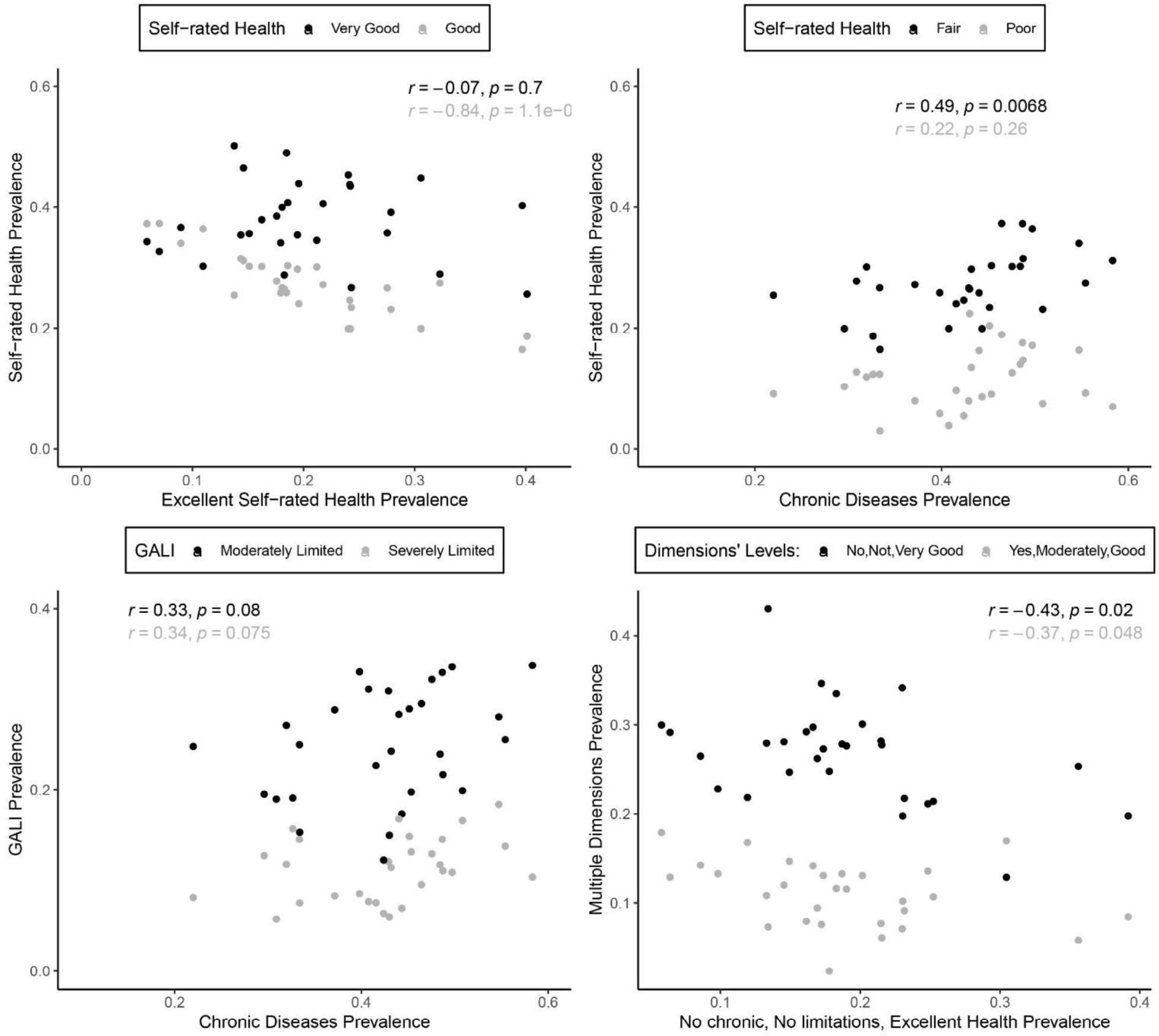

Notes: Standard Age= Eurostat for EU-28 population for 2018.
Source: Authors' estimations based on Eurostat (2021c).

Table A3: Well-being weights for health states in single dimensions in 29 European countries, 2018, by sex

A) Chronic diseases =YES

	Men				Women			
		Threshold				Threshold		
Country	Coefficient	1	10	Weight	Coefficient	1	10	Weight
Austria	-0.57	-3.06	0.71	0.85	-0.54	-3.05	0.62	0.85
Belgium	-0.57	-3.02	1.53	0.87	-0.66	-3.17	1.56	0.86
Bulgaria	-0.61	-2.04	1.66	0.84	-0.65	-2.11	1.65	0.83
Switzerland	-0.26	-2.78	0.77	0.93	-0.27	-2.76	0.67	0.92
Cyprus	-0.39	-2.41	1.15	0.89	-0.51	-2.52	1.17	0.86
Czech Rep.	-0.48	-2.79	1.02	0.88	-0.51	-2.84	0.97	0.87
Denmark	-0.56	-2.70	0.51	0.83	-0.60	-2.67	0.46	0.81
Estonia	-0.60	-2.90	1.24	0.86	-0.55	-2.87	1.10	0.86
Greece	-0.41	-2.20	1.45	0.89	-0.49	-2.24	1.45	0.87
Spain	-0.51	-2.83	1.22	0.87	-0.61	-2.90	1.19	0.85
Finland	-0.37	-3.00	1.10	0.91	-0.38	-3.21	1.09	0.91
France	-0.46	-2.92	1.29	0.89	-0.48	-2.82	1.33	0.89
Croatia	-0.64	-2.34	1.19	0.82	-0.67	-2.35	1.17	0.81
Hungary	-0.59	-2.72	1.52	0.86	-0.64	-2.71	1.50	0.85
Ireland	-0.45	-3.10	0.57	0.88	-0.49	-2.94	0.53	0.86
Italy	-0.42	-2.62	1.30	0.89	-0.48	-2.40	1.30	0.87
Lithuania	-0.58	-2.34	1.13	0.83	-0.65	-2.37	1.26	0.82
Luxembourg	-0.28	-2.53	1.17	0.92	-0.44	-2.87	1.11	0.89
Latvia	-0.74	-2.88	1.21	0.82	-0.69	-2.92	1.29	0.84
Netherlands	-0.54	-2.98	1.35	0.88	-0.53	-3.11	1.28	0.88
Norway	-0.47	-2.88	0.68	0.87	-0.48	-2.95	0.59	0.86
Poland	-0.33	-3.09	0.81	0.91	-0.40	-3.05	0.82	0.90
Portugal	-0.50	-2.41	1.11	0.86	-0.52	-2.26	1.11	0.85
Romania	-0.69	-2.77	1.29	0.83	-0.74	-2.76	1.31	0.82
Serbia	-0.48	-2.46	1.84	0.89	-0.56	-2.49	1.83	0.87
Sweden	-0.46	-2.63	0.73	0.86	-0.44	-2.86	0.62	0.87
Slovenia	-0.57	-2.59	1.12	0.85	-0.51	-2.62	1.07	0.86
Slovakia	-0.66	-2.50	0.87	0.80	-0.70	-2.60	0.87	0.80
UK	-0.30	-2.66	0.85	0.92	-0.35	-2.62	0.83	0.90

Notes: Controlled for age and age^2. Countries sorted in alphabetic order.
Source: Authors' estimations based on Eurostat (2021c).

Table A3 (cont.): Well-being weights for health states in single dimensions in 29 European countries, 2018, by sex B) Limitations of activities of daily living

	Men						Women					
	Coefficient		Threshold		Weight		Coefficient		Threshold		Weight	
Country	Moderate	Severe	1	10	Moderate	Severe	Moderate	Severe	1	10	Moderate	Severe
Austria	-0.56	-1.18	-3.23	0.71	0.86	0.70	-0.54	-1.11	-3.22	0.60	0.86	0.71
Belgium	-0.53	-0.96	-3.11	1.52	0.89	0.79	-0.55	-1.12	-3.32	1.57	0.89	0.77
Bulgaria	-0.61	-1.36	-2.05	1.69	0.84	0.64	-0.68	-1.28	-2.13	1.68	0.82	0.66
Switzerland	-0.31	-1.03	-2.90	0.76	0.91	0.72	-0.31	-0.95	-2.90	0.65	0.91	0.73
Cyprus	-0.43	-0.97	-2.47	1.19	0.88	0.73	-0.52	-1.05	-2.55	1.23	0.86	0.72
Czech Rep.	-0.47	-1.03	-2.88	1.04	0.88	0.74	-0.50	-1.04	-2.95	0.99	0.87	0.74
Denmark	-0.60	-1.40	-2.92	0.49	0.83	0.59	-0.61	-1.42	-2.89	0.44	0.82	0.57
Estonia	-0.56	-1.07	-2.99	1.28	0.87	0.75	-0.54	-0.91	-2.97	1.11	0.87	0.78
Greece	-0.28	-0.67	-2.23	1.45	0.92	0.82	-0.38	-0.74	-2.28	1.44	0.90	0.80
Spain	-0.62	-1.28	-2.92	1.26	0.85	0.69	-0.67	-1.38	-3.02	1.24	0.84	0.68
Finland	-0.52	-1.21	-3.21	1.11	0.88	0.72	-0.50	-1.10	-3.40	1.10	0.89	0.75
France	-0.44	-0.97	-3.00	1.34	0.90	0.78	-0.49	-0.94	-2.89	1.37	0.89	0.78
Croatia	-0.59	-1.23	-2.48	1.19	0.84	0.66	-0.64	-1.21	-2.48	1.18	0.83	0.67
Hungary	-0.63	-1.26	-2.82	1.57	0.86	0.71	-0.63	-1.31	-2.83	1.57	0.86	0.70
Ireland	-0.60	-0.86	-3.19	0.59	0.84	0.77	-0.49	-1.01	-3.01	0.57	0.86	0.72
Italy	-0.36	-0.94	-2.72	1.27	0.91	0.76	-0.40	-0.99	-2.52	1.26	0.89	0.74
Lithuania	-0.56	-1.24	-2.39	1.15	0.84	0.65	-0.62	-1.14	-2.38	1.31	0.83	0.69
Luxembourg	-0.26	-0.70	-2.62	1.15	0.93	0.82	-0.33	-0.80	-2.96	1.10	0.92	0.80
Latvia	-0.58	-1.06	-2.86	1.24	0.86	0.74	-0.56	-1.03	-2.93	1.31	0.87	0.76
Netherlands	-0.52	-1.32	-3.18	1.35	0.88	0.71	-0.54	-1.42	-3.44	1.26	0.88	0.70
Norway	-0.62	-1.44	-2.98	0.74	0.83	0.61	-0.57	-1.05	-3.00	0.64	0.84	0.71
Poland	-0.33	-0.68	-3.13	0.83	0.92	0.83	-0.38	-0.67	-3.03	0.87	0.90	0.83
Portugal	-0.54	-1.01	-2.49	1.12	0.85	0.72	-0.54	-1.05	-2.35	1.12	0.84	0.70
Romania	-0.43	-1.20	-2.84	1.27	0.90	0.71	-0.57	-1.16	-2.86	1.27	0.86	0.72
Serbia	-0.73	-0.97	-2.44	1.90	0.83	0.78	-0.69	-1.01	-2.43	1.92	0.84	0.77
Sweden	-0.59	-1.07	-2.57	0.80	0.83	0.68	-0.77	-0.87	-2.88	0.69	0.78	0.76
Slovenia	-0.56	-1.07	-2.69	1.12	0.85	0.72	-0.54	-1.13	-2.79	1.06	0.86	0.71
Slovakia	-0.43	-1.11	-2.57	0.87	0.88	0.68	-0.49	-1.15	-2.68	0.88	0.86	0.68
UK	-0.27	-0.89	-2.83	0.86	0.93	0.76	-0.28	-0.84	-2.73	0.85	0.92	0.76

Source: Authors' estimations based on Eurostat (2021c).

Table A3(cont.): Well-being weights for health states in single dimensions in 29 European countries, 2018, men

C) Self-rated health, men

	Coefficient				Threshold		Weight			
Country	Very good	Good	Fair	Poor	1	10	Very good	Good	Fair	Poor
Austria	-0.43	-1.05	-1.73	-2.06	-3.74	0.48	0.90	0.75	0.59	0.51
Belgium	-0.29	-0.79	-1.36	-1.97	-3.50	1.36	0.94	0.84	0.72	0.59
Bulgaria	-0.52	-0.92	-1.55	-2.27	-2.68	1.27	0.87	0.77	0.61	0.43
Switzerland	-0.32	-0.88	-1.41	-1.74	-3.20	0.59	0.92	0.77	0.63	0.54
Cyprus	-0.29	-0.66	-1.21	-1.62	-2.65	1.07	0.92	0.82	0.67	0.56
Czech Rep.	-0.49	-0.83	-1.50	-1.95	-3.46	0.65	0.88	0.80	0.64	0.53
Denmark	-0.46	-1.07	-1.78	-2.15	-3.41	0.16	0.87	0.70	0.50	0.40
Estonia	-0.52	-1.04	-1.75	-2.28	-3.71	0.79	0.88	0.77	0.61	0.49
Greece	-0.30	-0.49	-0.77	-1.27	-2.41	1.33	0.92	0.87	0.79	0.66
Spain	-0.46	-0.93	-1.57	-2.26	-3.44	0.93	0.90	0.79	0.64	0.48
Finland	-0.51	-1.17	-2.15	-2.16	-3.89	0.73	0.89	0.75	0.53	0.53
France	-0.50	-0.90	-1.55	-2.32	-3.59	0.96	0.89	0.80	0.66	0.49
Croatia	-0.54	-0.88	-1.48	-2.16	-3.04	0.81	0.86	0.77	0.62	0.44
Hungary	-0.45	-0.89	-1.64	-2.13	-3.43	1.19	0.90	0.81	0.64	0.54
Ireland	-0.36	-0.90	-1.53	-1.84	-3.52	0.39	0.91	0.77	0.61	0.53
Italy	-0.63	-0.79	-1.36	-2.14	-3.29	0.79	0.85	0.81	0.67	0.48
Lithuania	-0.71	-1.24	-1.85	-2.29	-3.32	0.39	0.81	0.67	0.50	0.38
Luxembourg	-0.32	-0.59	-0.99	-2.04	-3.05	0.91	0.92	0.85	0.75	0.48
Latvia	-0.54	-1.16	-1.75	-1.99	-3.65	0.65	0.87	0.73	0.59	0.54
Netherlands	-0.47	-1.17	-1.85	-2.50	-3.74	1.06	0.90	0.76	0.61	0.48
Norway	-0.49	-0.99	-1.69	-2.05	-3.46	0.41	0.87	0.74	0.56	0.47
Poland	-0.39	-0.69	-1.09	-1.40	-3.64	0.45	0.90	0.83	0.73	0.66
Portugal	-0.42	-0.88	-1.46	-1.98	-3.08	0.67	0.89	0.77	0.61	0.47
Romania	-0.32	-0.73	-1.44	-2.16	-3.19	1.05	0.92	0.83	0.66	0.49
Serbia	-0.68	-0.92	-1.47	-2.06	-3.21	1.42	0.85	0.80	0.68	0.55
Sweden	-0.53	-1.12	-1.80	-1.91	-3.24	0.43	0.86	0.70	0.51	0.48
Slovenia	-0.54	-1.09	-1.71	-1.73	-3.26	0.76	0.87	0.73	0.57	0.57
Slovakia	-0.48	-0.80	-1.32	-1.86	-2.99	0.56	0.87	0.78	0.63	0.47
UK	-0.20	-0.57	-1.14	-1.70	-3.07	0.71	0.95	0.85	0.70	0.55

Notes: Controlled for age and age^2. Countries sorted in alphabetic order.

Source: Authors' estimations based on Eurostat (2021c).

Table A3(cont.): Well-being weights for health states in single dimensions in 29 European countries, 2018, women

C) Self-rated health, women

	Coefficient				Threshold		Weight			
Country	Very good	Good	Fair	Poor	1	10	Very good	Good	Fair	Poor
Austria	-0.47	-1.04	-1.56	-1.98	-3.68	0.36	0.88	0.74	0.61	0.51
Belgium	-0.37	-0.94	-1.50	-1.87	-3.77	1.34	0.93	0.82	0.71	0.63
Bulgaria	-0.63	-1.11	-1.76	-2.49	-2.96	1.13	0.85	0.73	0.57	0.39
Switzerland	-0.33	-0.77	-1.50	-2.04	-3.27	0.48	0.91	0.80	0.60	0.46
Cyprus	-0.32	-0.74	-1.40	-1.50	-2.77	1.09	0.92	0.81	0.64	0.61
Czech Rep.	-0.41	-0.81	-1.48	-2.21	-3.50	0.63	0.90	0.80	0.64	0.46
Denmark	-0.36	-0.90	-1.73	-2.13	-3.32	0.18	0.90	0.74	0.50	0.39
Estonia	-0.43	-0.96	-1.40	-1.92	-3.54	0.68	0.90	0.77	0.67	0.55
Greece	-0.33	-0.60	-0.90	-1.33	-2.49	1.30	0.91	0.84	0.76	0.65
Spain	-0.46	-0.96	-1.63	-2.26	-3.55	0.89	0.90	0.78	0.63	0.49
Finland	-0.49	-1.08	-1.86	-2.57	-4.05	0.76	0.90	0.77	0.61	0.47
France	-0.49	-0.95	-1.60	-2.26	-3.50	0.98	0.89	0.79	0.64	0.50
Croatia	-0.52	-0.83	-1.54	-2.18	-3.06	0.80	0.87	0.78	0.60	0.44
Hungary	-0.49	-0.97	-1.65	-2.40	-3.54	1.11	0.90	0.79	0.65	0.48
Ireland	-0.35	-0.85	-1.65	-2.21	-3.38	0.39	0.91	0.77	0.56	0.42
Italy	-0.63	-0.87	-1.44	-2.13	-3.11	0.76	0.84	0.77	0.63	0.45
Lithuania	-0.66	-1.32	-1.92	-2.37	-3.41	0.49	0.83	0.66	0.51	0.39
Luxembourg	-0.35	-0.63	-1.17	-2.10	-3.42	0.85	0.92	0.85	0.72	0.51
Latvia	-0.57	-1.16	-1.73	-2.38	-3.85	0.65	0.87	0.74	0.62	0.47
Netherlands	-0.53	-1.23	-1.94	-2.77	-4.02	0.92	0.89	0.75	0.61	0.44
Norway	-0.44	-0.94	-1.37	-1.92	-3.44	0.34	0.88	0.75	0.64	0.49
Poland	-0.39	-0.74	-1.12	-1.46	-3.57	0.45	0.90	0.82	0.72	0.64
Portugal	-0.36	-0.86	-1.47	-1.94	-2.95	0.67	0.90	0.76	0.60	0.46
Romania	-0.37	-0.85	-1.48	-1.91	-3.25	1.01	0.91	0.80	0.65	0.55
Serbia	-0.73	-1.02	-1.60	-2.36	-3.39	1.35	0.85	0.79	0.66	0.50
Sweden	-0.56	-1.06	-1.60	-1.70	-3.45	0.29	0.85	0.72	0.57	0.55
Slovenia	-0.57	-1.09	-1.66	-2.02	-3.40	0.62	0.86	0.73	0.59	0.50
Slovakia	-0.59	-0.94	-1.48	-2.04	-3.22	0.47	0.84	0.75	0.60	0.45
UK	-0.27	-0.61	-1.22	-1.70	-3.03	0.68	0.93	0.83	0.67	0.54

Notes: Controlled for age and age^2. Countries sorted in alphabetic order.

Source: Authors' estimations based on Eurostat (2021c).

Table A4: Model coefficients, minimum and maximum thresholds, and well-being weights for health states in multiple dimensions in 29 European countries, by sex, 2018

No chronic diseases, no limitations, good self-rated health								
	Men				Women			
		Threshold				Threshold		
	Coefficient	1	10	Weight	Coefficient	1	10	Weight
Austria	-1.05	-3.79	0.47	0.75	-0.96	-3.70	0.36	0.76
Belgium	-0.75	-3.50	1.37	0.85	-0.99	-3.82	1.33	0.81
Bulgaria	-0.96	-2.69	1.27	0.76	-1.10	-2.98	1.13	0.73
Switzerland	-0.72	-3.23	0.60	0.81	-0.66	-3.32	0.48	0.83
Cyprus	-0.58	-2.66	1.07	0.85	-0.60	-2.81	1.08	0.84
Czech Rep.	-0.84	-3.47	0.65	0.80	-0.77	-3.54	0.63	0.81
Denmark	-1.00	-3.54	0.15	0.73	-0.84	-3.43	0.15	0.76
Estonia	-0.91	-3.73	0.79	0.80	-0.89	-3.57	0.69	0.79
Greece	-0.49	-2.42	1.34	0.87	-0.59	-2.50	1.30	0.84
Spain	-0.88	-3.47	0.93	0.80	-0.84	-3.58	0.89	0.81
Finland	-1.17	-3.94	0.76	0.75	-1.13	-4.11	0.76	0.77
France	-0.89	-3.64	0.96	0.81	-0.92	-3.52	0.98	0.80
Croatia	-0.88	-3.06	0.82	0.77	-0.88	-3.10	0.80	0.77
Hungary	-0.91	-3.48	1.18	0.80	-0.95	-3.60	1.09	0.80
Ireland	-0.80	-3.53	0.42	0.80	-0.76	-3.43	0.39	0.80
Italy	-0.63	-3.32	0.79	0.85	-0.71	-3.14	0.75	0.82
Lithuania	-1.23	-3.35	0.39	0.67	-1.40	-3.51	0.42	0.64
Luxembourg	-0.53	-3.05	0.95	0.87	-0.64	-3.47	0.85	0.85
Latvia	-1.18	-3.72	0.61	0.73	-1.09	-3.86	0.65	0.76
Netherlands	-1.43	-3.84	1.04	0.71	-1.27	-4.12	0.91	0.75
Norway	-0.79	-3.53	0.40	0.80	-0.97	-3.49	0.33	0.75
Poland	-0.80	-3.65	0.44	0.81	-0.79	-3.60	0.44	0.80
Portugal	-0.84	-3.14	0.65	0.78	-0.80	-2.98	0.67	0.78
Romania	-0.69	-3.21	1.06	0.84	-0.73	-3.30	1.00	0.83
Serbia	-0.99	-3.27	1.42	0.79	-1.12	-3.40	1.35	0.76
Sweden	-1.07	-3.26	0.43	0.71	-0.93	-3.48	0.31	0.76
Slovenia	-1.11	-3.29	0.76	0.73	-1.03	-3.48	0.60	0.75
Slovakia	-0.80	-3.01	0.55	0.78	-0.92	-3.26	0.46	0.75
UK	-0.63	-3.09	0.73	0.84	-0.63	-3.06	0.67	0.83

Notes: Health states in the multiple dimensions (chronic diseases, activity limitations, self-rated health) presented in the table are those with mean prevalence higher than 0.03; Controlled for age and age^2. Countries in alphabetic order
Source: Authors' estimations based on Eurostat (2021c).

Table A4 (cont.) : Model coefficients, minimum and maximum thresholds, and well-being weights for health states in multiple dimensions in 29 European countries, by sex, 2018

Chronic diseases, moderate limitations, good self-rated health								
	Men				Women			
		Threshold				Threshold		
	Coefficient	1	10	Weight	Coefficient	1	10	Weight
Austria	-1.14	-3.79	0.47	0.73	-1.05	-3.70	0.36	0.74
Belgium	-0.80	-3.50	1.37	0.84	-0.90	-3.82	1.33	0.83
Bulgaria	-0.94	-2.69	1.27	0.76	-1.15	-2.98	1.13	0.72
Switzerland	-0.80	-3.23	0.60	0.79	-0.77	-3.32	0.48	0.80
Cyprus	-0.63	-2.66	1.07	0.83	-0.71	-2.81	1.08	0.82
Czech Rep.	-0.87	-3.47	0.65	0.79	-0.89	-3.54	0.63	0.79
Denmark	-1.15	-3.54	0.15	0.69	-0.98	-3.43	0.15	0.73
Estonia	-1.12	-3.73	0.79	0.75	-1.06	-3.57	0.69	0.75
Greece	-0.45	-2.42	1.34	0.88	-0.56	-2.50	1.30	0.85
Spain	-1.00	-3.47	0.93	0.77	-1.06	-3.58	0.89	0.76
Finland	-1.19	-3.94	0.76	0.75	-1.08	-4.11	0.76	0.78
France	-0.95	-3.64	0.96	0.79	-1.00	-3.52	0.98	0.78
Croatia	-0.94	-3.06	0.82	0.76	-0.95	-3.10	0.80	0.75
Hungary	-1.00	-3.48	1.18	0.78	-1.07	-3.60	1.09	0.77
Ireland	-1.05	-3.53	0.42	0.73	-0.90	-3.43	0.39	0.76
Italy	-0.80	-3.32	0.79	0.81	-0.93	-3.14	0.75	0.76
Lithuania	-1.28	-3.35	0.39	0.66	-1.46	-3.51	0.42	0.63
Luxembourg	-0.54	-3.05	0.95	0.87	-0.66	-3.47	0.85	0.85
Latvia	-1.29	-3.72	0.61	0.70	-1.22	-3.86	0.65	0.73
Netherlands	-1.14	-3.84	1.04	0.77	-1.21	-4.12	0.91	0.76
Norway	-1.06	-3.53	0.40	0.73	-1.11	-3.49	0.33	0.71
Poland	-0.70	-3.65	0.44	0.83	-0.76	-3.60	0.44	0.81
Portugal	-1.03	-3.14	0.65	0.73	-0.95	-2.98	0.67	0.74
Romania	-0.79	-3.21	1.06	0.82	-0.97	-3.30	1.00	0.77
Serbia	-0.87	-3.27	1.42	0.81	-1.13	-3.40	1.35	0.76
Sweden	-1.12	-3.26	0.43	0.70	-1.32	-3.48	0.31	0.65
Slovenia	-1.09	-3.29	0.76	0.73	-1.13	-3.48	0.60	0.72
Slovakia	-0.78	-3.01	0.55	0.78	-0.98	-3.26	0.46	0.74
UK	-0.52	-3.09	0.73	0.86	-0.58	-3.06	0.67	0.85

Notes: Health states in the multiple dimensions (chronic diseases, activity limitations, self-rated health) presented in the table are those with mean prevalence higher than 0.03. Controlled for age and age^2. Countries in alphabetic order
Source: Authors' estimations based on Eurostat (2021c).

Table A5: Life expectancy (LE), disability adjusted health expectancy (DALE)*, health expectancy (HE) and well-being adjusted health expectancy (WAHE) at age 15 for the three dimensions of health, men, 2018

			HE				WAHE			
Country	LE	DALE	Chronic	GALI	Self-rated	Multi.	Chronic	GALI	Self-rated	Multi.
Latvia	55.5	49.2	38.3	38.2	6.3	6.2	52.4	52.6	45.0	44.5
Lithuania	56.4	49.0	40.4	42.9	6.8	6.6	53.7	53.8	42.2	41.1
Bulgaria	57.2	48.3	47.2	50.5	16.2	16.0	55.5	55.8	49.5	49.5
Romania	57.4	50.0	49.1	45.7	19.9	19.1	56.0	55.7	52.8	52.9
Hungary	58.1	50.2	39.7	46.6	13.8	13.2	55.5	56.0	51.1	50.6
Serbia	59.0	53.5	43.9	53.1	16.0	15.7	57.3	57.9	50.6	50.5
Poland	59.2	51.1	39.6	47.3	14.0	13.2	57.5	57.8	52.7	52.4
Estonia	59.3	56.3	33.4	38.7	6.7	6.1	55.5	55.8	48.9	49.0
Slovakia	59.5	51.2	42.9	42.2	15.8	15.2	56.2	56.2	50.7	50.2
Croatia	60.5	52.2	42.0	42.4	22.8	21.8	57.1	56.7	51.9	51.9
Czechia	61.5	52.3	43.6	48.2	18.6	17.9	59.2	59.4	53.9	53.9
Portugal	63.7	54.9	42.1	47.5	10.0	8.9	60.7	60.8	53.4	53.1
Slovenia	63.8	53.9	40.4	42.8	15.9	14.8	60.2	60.0	53.6	53.7
Finland	64.3	54.9	36.3	45.1	12.9	11.0	61.8	61.3	55.0	55.3
Denmark	64.5	55.4	46.6	47.2	17.2	16.1	61.3	60.7	54.4	54.0
Greece	64.7	55.1	51.3	51.3	33.8	33.1	63.2	63.1	61.0	61.0
Austria	64.8	55.3	42.9	44.0	22.7	20.3	61.5	61.0	57.1	57.1
Belgium	64.8	54.7	50.2	50.1	21.1	19.7	63.0	62.7	60.1	60.2
United Kingdom	64.9	54.6	38.1	47.9	21.2	18.7	62.6	62.4	59.9	60.1
France	65.2	56.4	40.8	49.8	16.7	14.8	62.5	62.9	57.4	57.1
Luxembourg	65.6	56.3	49.7	48.5	16.2	15.2	64.4	63.8	59.4	59.9
Netherlands	65.7	56.4	45.5	46.6	17.7	16.2	63.2	62.8	58.2	56.9
Ireland	65.7	55.8	47.2	54.4	30.3	27.4	63.5	63.7	60.5	60.8
Spain	66.0	56.7	45.9	54.0	17.3	16.3	63.4	63.8	58.5	58.5
Sweden	66.1	57.0	44.8	59.4	20.2	18.0	63.2	64.7	56.2	56.0
Cyprus	66.2	55.4	37.9	48.5	28.5	26.9	63.1	63.4	61.0	61.1
Norway	66.4	56.3	43.9	56.7	18.5	16.4	63.4	64.0	57.5	56.8
Italy	66.5	56.3	58.3	52.8	12.2	11.9	65.6	64.9	57.0	56.8
Switzerland	67.3	57.3	45.3	48.4	25.1	21.6	65.6	65.0	61.5	61.6

Notes: * as explained in the text, we use the old name, DALE, for the indicator, the new name is HALE; countries ordered by LE;
Source: Authors' estimations based on Eurostat (2021c), Eurostat (2021) and GBD (2020).

Table A6: Life expectancy (LE), disability adjusted health expectancy (DALE)*, health expectancy (HE) and well-being adjusted health expectancy (WAHE) at age 15 for the three dimensions of health, women, 2018

			HE				WAHE			
Country	LE	DALE	Chronic	GALI	Self-rated	Multi.	Chronic	GALI	Self-rated	Multi.
Serbia	63.9	50.9	43.6	55.8	15.2	14.8	61.3	62.5	53.3	53.3
Bulgaria	64.2	53.2	50.2	54.0	14.7	14.4	61.8	62.1	53.1	53.1
Romania	64.8	55.0	50.9	46.2	17.8	16.9	62.3	61.6	57.5	57.3
Hungary	64.9	54.7	40.1	48.5	12.6	12.2	61.2	61.8	55.4	54.9
Latvia	65.1	54.8	39.8	40.5	4.9	4.8	61.0	61.2	51.7	51.7
Lithuania	66.1	55.2	41.8	46.2	5.9	5.4	61.8	62.2	48.6	46.5
Slovakia	66.3	55.7	43.9	43.4	15.0	14.6	61.7	61.8	54.1	53.5
Croatia	66.8	55.9	43.1	44.7	20.3	19.3	62.3	61.9	56.0	55.7
Poland	67.1	56.7	40.1	50.6	11.9	11.2	64.3	65.1	58.4	57.9
Czechia	67.3	56.0	43.8	49.0	16.3	15.7	64.2	64.3	58.6	58.6
Estonia	67.9	51.0	35.9	41.6	7.5	7.2	63.5	63.6	56.6	56.6
Denmark	68.3	56.1	44.6	44.6	15.8	14.2	63.7	62.7	57.5	56.8
United Kingdom	68.4	55.6	37.2	46.7	21.2	18.2	65.3	65.3	61.9	61.8
Netherlands	68.7	56.5	43.9	43.5	14.2	13.0	65.8	64.9	59.5	58.0
Belgium	69.3	56.3	51.1	50.3	19.4	18.5	66.8	66.4	62.7	62.5
Austria	69.3	57.3	43.1	44.3	22.4	20.4	65.5	64.9	60.2	60.1
Ireland	69.4	56.5	49.3	56.3	30.2	27.6	66.5	67.0	63.6	63.6
Sweden	69.5	57.2	42.4	58.3	18.6	16.7	66.1	67.0	58.6	58.3
Slovenia	69.6	58.0	40.7	42.7	13.3	12.3	65.6	64.8	56.8	56.5
Norway	69.7	57.0	41.0	54.6	18.8	16.8	65.8	66.8	60.3	59.5
Finland	69.7	57.2	32.7	42.2	11.8	9.8	66.4	65.9	60.2	60.1
Greece	69.8	56.7	52.7	52.0	33.4	32.7	67.5	67.2	64.6	64.7
Portugal	69.9	57.1	38.9	43.3	9.2	8.3	65.1	64.9	56.5	56.4
Luxembourg	70.0	57.2	48.9	46.7	14.8	13.3	67.7	67.1	62.3	62.3
Cyprus	70.1	56.3	36.9	48.2	27.0	25.5	65.5	66.1	63.2	62.9
Italy	70.9	57.8	59.2	52.8	11.5	11.3	69.4	68.4	59.1	58.8
Switzerland	71.1	57.9	43.9	46.5	23.8	20.0	68.9	68.1	64.4	64.1
France	71.3	58.4	42.0	51.2	15.1	13.9	67.9	68.2	61.5	61.3
Spain	71.6	58.2	45.8	54.2	16.2	15.2	67.7	68.2	62.1	62.1

Notes: * as explained in the text, we use the old name, DALE, for the indicator, the new name is HALE; countries ordered by LE;
Source: Authors' estimations based on Eurostat (2021c), Eurostat (2021) and GBD (2020).

Figure A3: Bland-Altman plots for agreement between WAHE based on chronic morbidity and other summary measures of population health in 29 countries by sex, 2018.

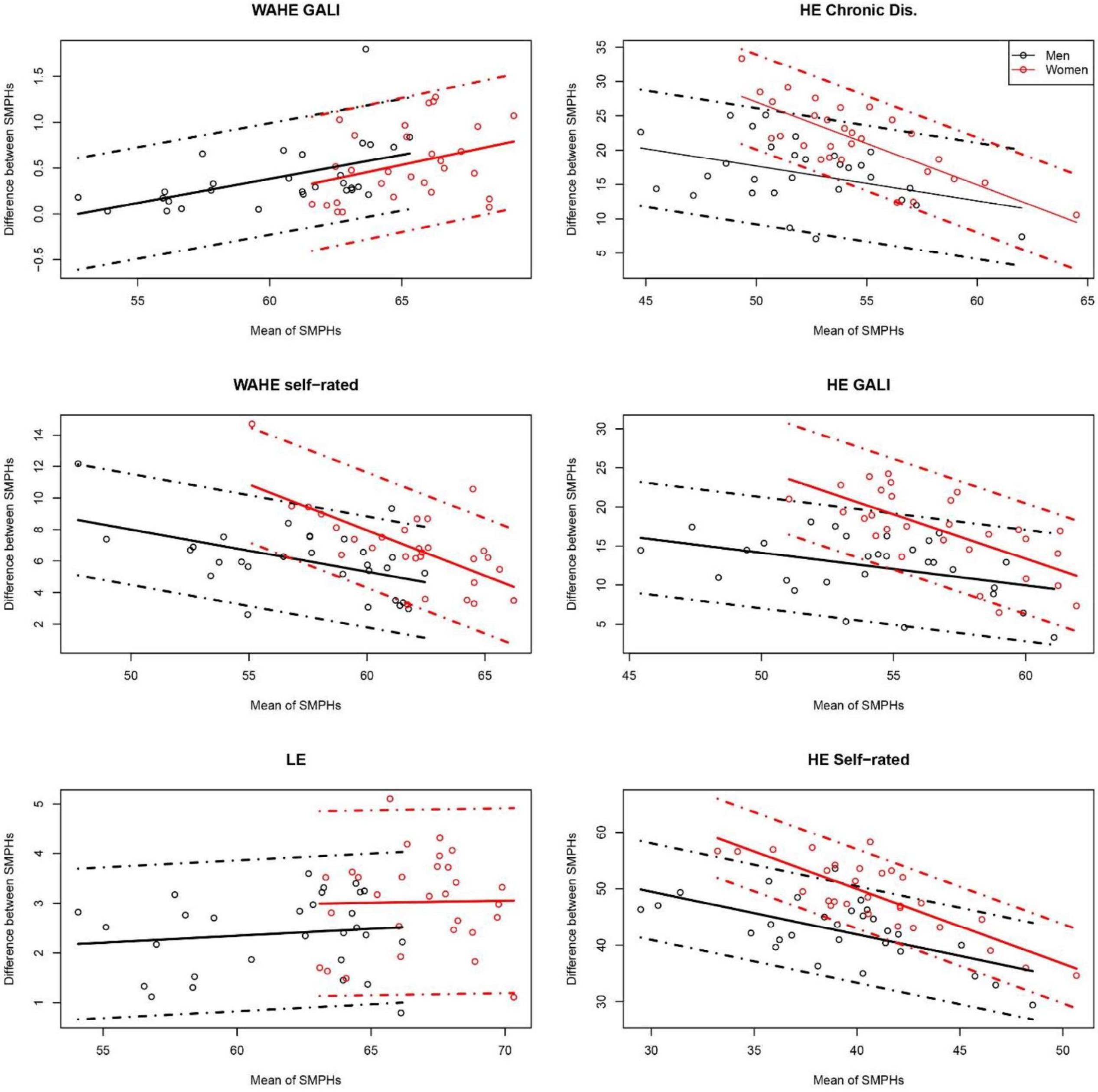

Note: Dots represent country observations; solid lines represent the linear model; dashed lines represent the 95% confidence intervals.
Source: Authors' estimations based on Eurostat (2021c), Eurostat (2021) and GBD (2020).

Figure A4: Bland-Altman plots for agreement between WAHE based on GALI and other summary measures of population health in 29 countries by sex, 2018.

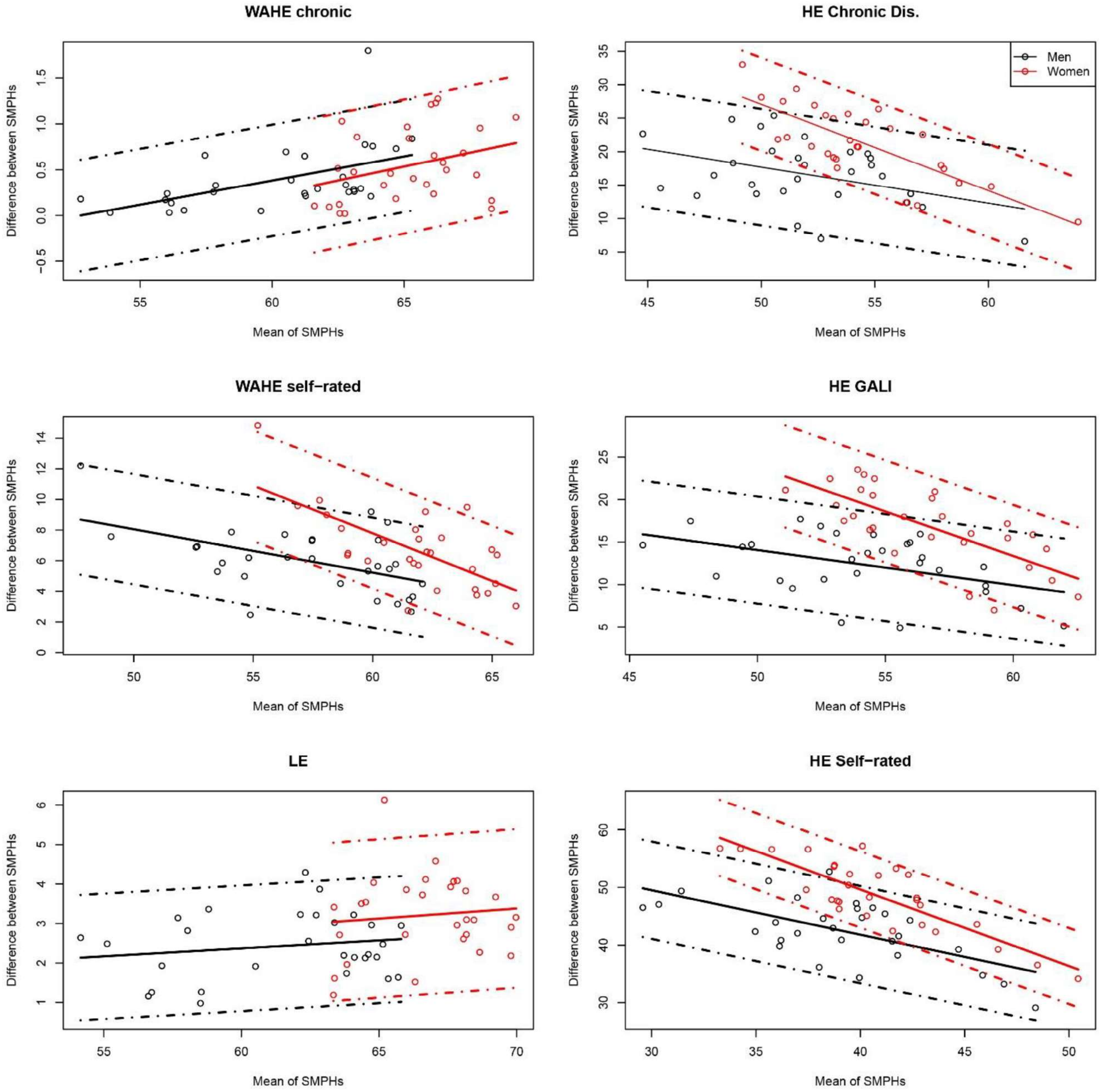

Note: Dots represent country observations; solid lines represent the linear model; dashed lines represent the 95% confidence intervals.
Source: Authors' estimations based on Eurostat (2021c), Eurostat (2021) and GBD (2020).
