# Supplementary material for: Well-Being Adjusted Health Expectancy: A New Summary Measure of Population Health
Source: Eur J Popul. 2022 Aug 8;38(5):1009–31. doi: 10.1007/s10680-022-09628-1 (PMC9726765; doi:10.1007/s10680-022-09628-1)
Supplement: Supplementary file 2 — Supplementary file1 (PDF 1152 KB) [file 10680_2022_9628_MOESM2_ESM.pdf]

Online Supplementary Material 2: Additional Methods Descriptions

1. Ordered Probit Model for the Well-being Weights

Well-being is a latent continuous variable, denoted by WB^*. When answering the question "Overall, how satisfied are you with your life?" respondents assess their well-being using a discrete scale, ranging from 1 (maximum unsatisfied) to 10 (maximum satisfied). The collected responses measure observed well-being, which is denoted by WB^O.
Latent well-being is assumed to be related to health status as:

$$WB^* = \beta_0 + H\beta_1 + \boldsymbol{X\beta_2} + \varepsilon$$

where H is the health status of an individual; $\boldsymbol{X}$ is a vector of other individual characteristics; $\boldsymbol{\beta}$ are vectors of coefficients, and ε is a normal distributed random error term.

Since an ordered scale is applied to the well-being question, observed well-being (WB^O) is a categorical ordered response variable. It is assumed to be related to the latent well-being variable WB^*as:

$$WB^O = i \leftrightarrow c_{i-1} < WB^* \leq c_i, \qquad i = 1, \dots ,10$$

where *i* denotes response categories (from 1 to 10 for observed well-being in this study); c_i are threshold levels, where $c_0 = -\infty$ and $c_n = \infty$,and the remaining threshold levels are specified as:

$c_i = \delta_i$ for $i = 1, \dots ,10$

where δ_i are coefficients to be estimated in the ordered link model (with the link being, e.g., logit or probit). While thresholds are constant in the ordered link models, generalised ordered models allow thresholds to be dependent on covariates (i.e., sex, age, country of residence) and, hence, they are modelled separately (Greene et al., 2014, King et al., 2004).

Following the notations above, the probability of observing an i level of well-being WB^O for individual j is given by:

$$\begin{aligned} P\left(WB_j^0 = i\right) &= P(c_{i-1} \leq WB^* \leq c_i) \\ &= P\left(c_{i-1} - \beta_0 + H_j\beta_1 + \boldsymbol{X_j\beta_2} \leq \varepsilon_j \leq c_i - \beta_0 + H_j\beta_1 + \boldsymbol{X_j\beta_2}\right) \\ &= F\left(c_i - \beta_0 + H_j\beta_1 + \boldsymbol{X_j\beta_2}\right) - F\left(c_{i-1} - \beta_0 + H_j\beta_1 + \boldsymbol{X_j\beta_2}\right) \end{aligned}$$

where F denotes the cumulative distribution function for ε_j, $c_0 = c_{i-1} - \infty$ and $c_n = \infty$.

In the logit link model, the cumulative distribution function for ε_j is specified by cumulative logistic distribution. In the probit link model, $\varepsilon_j$are assumed to be independent and follow a normal distribution with $E(\varepsilon_j) = 0$ and $D(\varepsilon_j) = \sigma$. Hence, based on the above probability for the probit link, we can specify the log-likelihood function as:

$$\log L = \sum_{j=1}^{N} \sum_{i=1}^{9} z_{ij} log[\Phi(c_i - \beta_0 + H_j\beta_1 + \boldsymbol{X_j\beta_2}) - \Phi(c_{i-1} - \beta_0 + H_j\beta_1 + \boldsymbol{X_j\beta_2})]$$

where $z_{ij} = 1$ if $WB_j^0 = i$ and 0 otherwise. Φ denotes a cumulative function of a normal distribution. The log-likelihood function is maximised to derive the estimators of coefficients $\boldsymbol{\beta}$ and the cutpoints for the latent distribution of well-being c_i are derived.

Well-being weights for each decreased health state are equal to the respective, normalised regression coefficient (β_1). In the normalisation used in this paper, we follow the approach by Cutler et al. (1997), who estimated QALY from a normal ordered probit model as a proportional decrease in self-rated health resulting from a health limitation. QALY weights for disease i are calculated as:

$$QALY_i = \frac{\hat{\beta}_i}{\delta_1 - \delta_n}$$

where $\hat{\beta}_i$ is the coefficient for the disease i and δ_1, δ_n are the lowest and the highest thresholds for the latent health variable from the estimated models.

An alternative method to standardized coefficients, based on the proposed by Dooslaer and Jones (2003,p.65) solutions for self-rated health weights, is to estimate predicted values of respondents´ well-being based on the model and dividing the model coefficients by the difference between the maximum and the minimum individual predictions (a similar solution was proposed by Jürges (2007) and Oksuzyan et al. (2019)).

Generalised ordered link models are potentially an alternative to the estimation of separate models for the sub-groups of populations. Generalised ordered link models allow the thresholds to be dependent on characteristics that the sub-groups are specified upon (e.g., sex, age, country of residence) (Greene et al., 2014, King et al., 2004). This approach, however, does not allow for the differentiation of the effect of decreased health on well-being across the study contextual characteristics (Oksuzyan, 2019; Rebelo and Pereira, 2014), which is the most important feature of WAHE, and a limitation of other SMPH.

2. Bland-Altman Plots with a Linear Trend to Assess Agreement between WAHE and Other SMPHs

Following the guidelines developed by Kottner et al. (2011) for reporting reliability and agreement studies, Bland-Altman plots with limits of agreement are a recommended method for analysing the agreement of continuous variables.

In Bland-Altman plots the difference between two variables for each observation point is plotted against the mean. If the differences are normally distributed, $(1-\alpha)\%$ of the observations will lie between the limits of agreement of $\bar{d} - z_\alpha s$ and $\bar{d} + z_\alpha s$, where $\bar{d}$ is the mean and s is the standard deviation of differences between variables, and z_α is the upper $\left(1 - \frac{\propto}{2}\right)$ critical value for the standard normal distribution (Bland and Altman, 1986).

In this study, a linear regression model with the dependent variable being the difference between the measurements and the independent variable being the mean of the measurements can be fitted. Assuming that the residuals follow a normal distribution with mean zero and variance σ^2, the expected value of the difference between the methods can be estimated as:

$$\hat{d} = b_0 + b_1 a.$$

In this case, the $(1-\alpha)$ limits of agreement are $\hat{d} \pm z_\alpha \sqrt{\frac{2}{\pi}} \hat{R}$, where $\hat{R} = c_0 + c_1 a$ are the absolute values of residuals ($\hat{R} = d - \widehat{d)}$ regressed on the mean of the measurements (*a*). In case no significant relationship between $\hat{R}$ and *a* is observed, $\hat{R}$ is simply estimated as a standard deviation of the residuals (Bland and Altman, 1999).

3. Intraclass Correlation Coefficients to Assess Reliability of WAHE and Other SMPHs

Following the guidelines developed by Kottner et al. (2011) for reporting reliability and agreement studies, the intraclass correlation coefficient (ICC) is the preferred statistical method for analysing reliability for continuous variables.

For assessing the reliability of the SMPHs, we apply a two-way mixed effects model: The SMPHs are considered a fixed effect, because population health is only assessed by indices belonging to the group specified in this study. However, we are interested in the reliability of this given group and do not extend our results to any additional population health index. However, the list of countries can be extended, so countries are considered a random effect in our model. Next, we need to choose a type of measurement between the mean value of the indices and single indices measurement; in this study, we apply the former. Using the mean measurement across all SMPHs to study reliability allows us to answer the research question of whether this group of measures can reliably discern population health levels across the study

countries. Finally, by excluding the indices one by one and comparing the new ICC to that of the whole group, we examine whether incorporating this additional information to a given SMPH facilitates distinguishing between population health levels in the studied countries. The method described above used to derive ICC in this study is denoted by *ICC(C, k)*, as classified by McGraw and Wong (1996) and ICC(3,k), as classified by Shrout and Fleiss (1979). Regarding the relationship between these two standards of the classification, see also Koo and Li (2016). In Shrout and Fleiss' (1979) classification (1979), *ICC(3, k)* are applied to assess reliability in situations where, "each target is rated by each of the same k judges, who are the only judges of interest" (p.421). In our study "judges" denotes SMPHs and "targets" denotes countries. Hence, the value of index i (*i=1,2,..,k*) in the two-way mixed model for country *j* (*j=1,2,..,n*) is equal to:

$$x_{ij} = \mu + a_i + b_j + v_{ij}$$

where μ is the overall mean of all values of the indices across the countries; b_j is the difference from μ of the jth country true score (e.g., mean of all scores), and is assumed to be normally distributed with mean=0 and variance= σ_T^2; a_i is the distance in the mean of ith index from μ.

In the *ICC(3, k)* model:
$\sum_{i=1}^{k} a_i = 0$;

v_{ij} denotes the random error in the ith index for country *j*.

As demonstrated by Shrout and Fleiss (1979) and McGraw and Wong (1996), and based on the above model specification, the estimator for the ICC is:

$$\widehat{ICC}(3, k) = \frac{MS_R - MS_E}{MS_R}$$

where MS_R is the mean square of differences in the index values between the countries, and $MS_E$is the mean residual differences, i.e., the mean square error of measurement from the repeated measures analysis of variance (ANOVA).

References

Berger, N., Van Oyen, H., Cambois, E., Fouweather, T., Jagger, C., Nusselder, W., & Robine, J. M. (2015). Assessing the validity of the Global Activity Limitation Indicator in fourteen European countries. *BMC Medical Research Methodology*, *15*(1), 1-8.

Bland, J. M., & Altman, D. (1986). Statistical methods for assessing agreement between two methods of clinical measurement. *The Lancet*, *327*(8476), 307-310.

Bland, J. M., & Altman, D. G. (1999). Measuring agreement in method comparison studies. *Statistical Methods in Medical Research*, *8*(2), 135-160.

Cutler, D. M., Richardson, E., Keeler, T. E., & Staiger, D. (1997). Measuring the health of the US population. *Brookings Papers on Economic Activity. Microeconomics*, *1997*, 217-282.

Greene, W., Harris, M. N., Hollingsworth, B., & Weterings, T. A. (2014). Heterogeneity in ordered choice models: A review with applications to self-assessed health. *Journal of Economic Surveys*, *28*(1), 109-133.

Van Doorslaer, E., & Jones, A. M. (2003). Inequalities in self-reported health: validation of a new approach to measurement. *Journal of Health Economics*, 22(1), 61-87.

King, G., Murray, C. J., Salomon, J. A., & Tandon, A. (2004). Enhancing the validity and cross-cultural comparability of measurement in survey research. *American Political Science Review*, *98*(1), 191-207.

Koo, T. K., & Li, M. Y. (2016). A guideline of selecting and reporting intraclass correlation coefficients for reliability research. *Journal of Chiropractic Medicine*, *15*(2), 155-163.

Kottner, J., Audigé, L., Brorson, S., Donner, A., Gajewski, B. J., Hróbjartsson, A., C. Roberts, M. Shoukri, & Streiner, D. L. (2011). Guidelines for reporting reliability and agreement studies (GRRAS) were proposed. *International Journal of Nursing Studies*, *48*(6), 661-671.

McGraw, K. O., & Wong, S. P. (1996). Forming inferences about some intraclass correlation coefficients. *Psychological Methods*, *1*(1), 30.

Rebelo, L. P., & Pereira, N. S. (2014). Assessing health endowment, access and choice determinants: Impact on retired Europeans'(In) activity and quality of life. *Social Indicators Research*, 119(3), 1411-1446.

Shrout, P. E., & Fleiss, J. L. (1979). Intraclass correlations: uses in assessing rater reliability. *Psychological Bulletin*, *86*(2), 420.
